# Biochemical Characterization of Highly Purified Leucine-Rich Repeat Kinases 1 and 2 Demonstrates Formation of Homodimers

**DOI:** 10.1371/journal.pone.0043472

**Published:** 2012-08-29

**Authors:** Laura Civiero, Renée Vancraenenbroeck, Elisa Belluzzi, Alexandra Beilina, Evy Lobbestael, Lauran Reyniers, Fangye Gao, Ivan Micetic, Marc De Maeyer, Luigi Bubacco, Veerle Baekelandt, Mark R. Cookson, Elisa Greggio, Jean-Marc Taymans

**Affiliations:** 1 Department of Biology, University of Padova, Padova, Italy; 2 Laboratory for Neurobiology and Gene Therapy, Katholieke Universiteit Leuven, Leuven, Belgium; 3 Laboratory for Biomolecular Modelling, Katholieke Universiteit Leuven, Heverlee, Belgium; 4 Cell Biology and Gene Expression Unit, Laboratory of Neurogenetics, National Institute on Aging, Bethesda, Maryland, United States of America; Mount Sinai School of Medicine, United States of America

## Abstract

Leucine-rich repeat kinase 1 and 2 (LRRK1 and LRRK2) are large multidomain proteins containing kinase, GTPase and multiple protein-protein interaction domains, but only mutations in LRRK2 are linked to familial Parkinson's disease (PD). Independent studies suggest that LRRK2 exists in the cell as a complex compatible with the size of a dimer. However, whether this complex is truly a homodimer or a heterologous complex formed by monomeric LRRK2 with other proteins has not been definitively proven due to the limitations in obtaining highly pure proteins suitable for structural characterization. Here, we used stable expression of LRRK1 and LRRK2 in HEK293T cell lines to produce recombinant LRRK1 and LRRK2 proteins of greater than 90% purity. Both purified LRRKs are folded, with a predominantly alpha-helical secondary structure and are capable of binding GTP with similar affinity. Furthermore, recombinant LRRK2 exhibits robust autophosphorylation activity, phosphorylation of model peptides *in vitro* and ATP binding. In contrast, LRRK1 does not display significant autophosphorylation activity and fails to phosphorylate LRRK2 model substrates, although it does bind ATP. Using these biochemically validated proteins, we show that LRRK1 and LRRK2 are capable of forming homodimers as shown by single-particle transmission electron microscopy and immunogold labeling. These LRRK dimers display an elongated conformation with a mean particle size of 145 Å and 175 Å respectively, which is disrupted by addition of 6M guanidinium chloride. Immunogold staining revealed double-labeled particles also in the pathological LRRK2 mutant G2019S and artificial mutants disrupting GTPase and kinase activities, suggesting that point mutations do not hinder the dimeric conformation. Overall, our findings indicate for the first time that purified and active LRRK1 and LRRK2 can form dimers in their full-length conformation.

## Introduction

**Figure 1:**
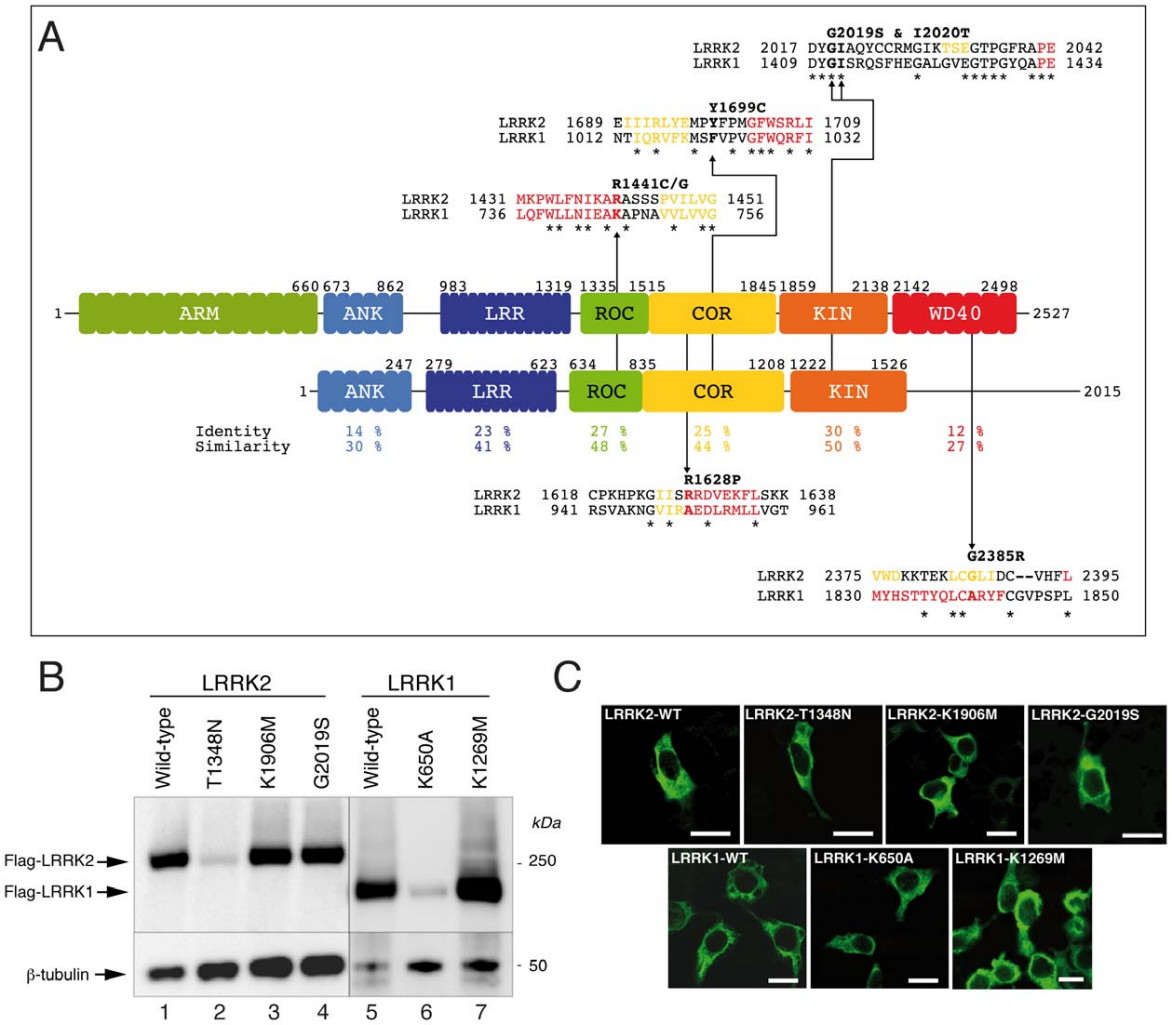
Characterization of HEK293T cell line stably expressing 3xFlag-LRRK1 and LRRK2. (A) Schematic alignment of LRRK1 and LRRK2. Predicted functional domains are drawn to scale at the relative location within the full protein sequence. For domains containing repeat sequences, predicted individual repeat units are depicted. The sequence identity and similarity for the LRR, ROC, COR and Kinase domains are given below the schematic. Also given are detailed alignments of LRRK1 and LRRK2 at the level of common LRRK2 clinical mutations. Abbreviations for the domains: ARM, armadillo repeat domain; ANK, ankyrin repeat domain; LRR, leucine rich repeat domain; ROC, Ras of comple proteins domain; COR, C-terminal of ROC domain; Kin, kinase domain; WD40, WD40 repeat domain. (B) Representative western blot analysis of HEK293T cells stably expressing (from lane 1 to 7) 3xFlag-tagged LRRK2 wild-type, T1348N GTP deficient binding mutant, K1906M kinase dead, G2019S pathogenic mutant and LRRK1 wild-type K650A GTP deficient binding mutant, K1269M kinase dead. Upper panel shows membranes probed with Flag (M2) antibody (note that LRRK2 and LRRK1 have different exposure time due to the very low expression of T1348N mutant). Lower panel shows β-tubulin loading control. (C) Representative confocal images of stable HEK293T cells expressing LRRK1 and LRRK2 wild-type and mutants. Scale bar 10 µm.

ROCO proteins are a family of multidomain proteins characterized by the presence of tandem ROC (Ras of Complex Proteins) and COR (C-terminal of ROC) domains [1], [2], [3]. There are four human ROCO proteins: Leucine-rich repeat kinase 1 and 2 (LRRK1 and LRRK2), death-associated kinase 1 (DAPK1) and Malignant fibrous histiocytoma amplified sequence 1 (MFASH1). LRRK1 and LRRK2 share a similar domain organization, which includes a serine-threonine kinase domain C-terminal of ROC-COR, and leucine-rich and ankyrin-like repeats at the N-terminus. The major differences between LRRK1 and LRRK2 are at the N-terminal region, where LRRK2 has a large number of unique repeats [2], [4], [5] and unique phosphorylated consensus binding sites for 14-3-3 [6], [7]. There are important differences also in the C-terminal regions [5] which show the lowest degree of homology compared to other domains [8] (Fig. 1). This divergence may be relevant with respect to a recently proposed pre-synaptic function of LRRK2, which was shown to interact with a number of pre-synaptic proteins *via* its C-terminal WD40 domain [9].

Previous studies have shown that both LRRK1 and LRRK2 bind GTP [10], [11], are basally phosphorylated in cells and share a similar cytoplasmic distribution [10]. However, only mutations in LRRK2 [12], [13], but not in LRRK1 [14], [15], have been identified as a cause of familial Parkinson's disease (PD).

Pathological LRRK2 mutations are clustered in the catalytic core of the protein (ROC-COR and kinase domains) suggesting that altered activity may be associated with disease. In support of this notion, *in vitro* studies have shown that mutations in the ROC/GTPase (R1441C/G) and COR (Y1996C) domains lower LRRK2 GTPase activity [16], [17], [18], while the common G2019S mutation in the kinase domain increases kinase activity by 2–3 fold (reviewed in [19]). It has been suggested that there may be an intramolecular regulation mechanism between ROC and kinase domains [20], [21], [22]. Although the molecular mechanisms remain to be clarified, this suggests that mutations with different effects on local protein structure might have common effects on overall function (reviewed in [23]).

LRRK1 and LRRK2 can form hetero- and homo-dimers [8], [24], [25], [26], [27] and it has been suggested that the dimer is the functional unit [27], [28]. The other human ROCO protein DAPK1 has also been recently shown to be a functional dimer [29]. Dimerization is an important process to control protein function and many proteins, including some classes of GTPases exert their physiological function as dimers [30]. Structures of the human ROC domain [31] and the *C. tepidum* bacterial ROC-COR bidomain [32] indicate that isolated portions of LRRK2 and homologues can form dimers. However, all these observations were made either from cell lysates or from only isolated domains only, and it is not clear if full-length proteins form dimers in the same manner. In cell lysates, LRRK2 is associated with a number of protein binding partners including 14-3-3 [6], [7], [33], tubulins [34], [35] and Hsp90 [36], [37], making assignment of homodimers compared to heterologous interactions difficult. Specific LRRK1 interactors such as Grb2 [38], [39] and Bcr-Abl [39], [40] have also been identified. Recently, Ito and Iwatsubo suggested that LRRK2 protein from cell lysates exists predominantly as a monomer [41]. Therefore, establishing whether the PD associated LRRK2 is a dimer in its active form and comparing its quaternary organization with its closest homolog LRRK1, is a crucial step to understand how these complex proteins are regulated and activated during signal transduction.

To address this we developed a heterologous mammalian expression system and affinity purification protocol to obtain highly purified, folded and functional LRRK1 and LRRK2 full-length proteins and used them to conduct the first characterization of their quaternary organization by imaging single molecules via transmission electron microscopy.

## Results

### Generation of HEK293T cells stably expressing full-length LRRK1 and LRRK2 using lentiviral vectors

Sequence analysis of LRRK1 and LRRK2 revealed an overall identity/similarity of 16/29%, whereas homology is higher in the LRR, ROC, COR and kinase domains than in other domains (Fig. 1A).

In order to characterize the biochemical properties and quaternary organization of LRRK1 and LRRK2, we generated lentiviral vectors for expression of 3xFlag full-length LRRK1 and LRRK2 in mammalian cells. Despite the large size of LRRK1 and LRRK2 (227 kDa and 286 kDa respectively), it was possible to generate functional LVs. We used these vectors to generate stable HEK293T lines expressing the two wild-type proteins and a set of mutants (Fig. 1B). Both expression levels and number of positive cells of LRRK1 wild-type and K1269M were significantly higher compared to LRRK2 proteins whereas mutants disrupting GTP binding (K650A for LRRK1 and T1348N for LRRK2) displayed significantly decreased steady state expression levels. The cellular distribution of all proteins was predominantly cytoplasmic (Fig. 1C). These results suggest that stable HEK293T lines represent an appropriate source of recombinant LRRK1 and LRRK2 proteins.

### Full-length LRRK1 and LRRK2 from HEK293T cells are folded

**Figure 2:**
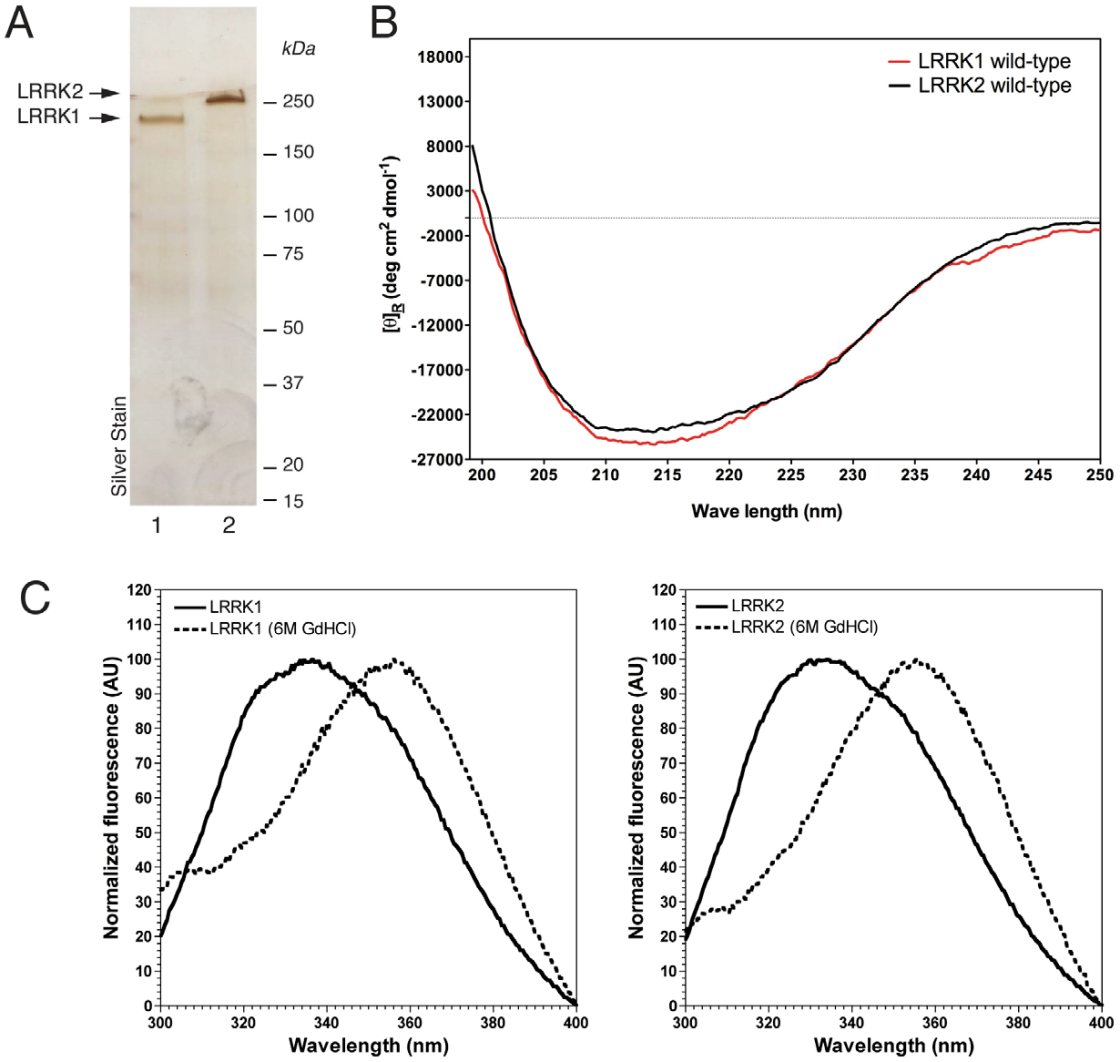
Purification of soluble full-length 3xFlag-LRRK1 and 3xFlag-LRRK2. (A) Representative silver staining of purified 3xFlag-LRRK1 and LRRK2 purification indicates highly pure protein fractions. Markers are in kilodaltons (B) Circular dichroism analysis of purified 3xFlag LRRK1 and LRRK2. Representative spectra are reported as mean residue molar ellipticity (deg cm^2^ dmol^−1^). (C) Representative fluorescence spectra of purified LRRK1 (right) and LRRK2 (left) before (solid line) and after (dashed line) addition of 6M GdHCl using an excitation wavelength of 280 nm. Fluorescence intensity was normalized to the highest peak.

Previous studies have employed full-length LRRK2 protein in solution for biochemical testing [49], [50], [51], however these proteins were purified using large GST tags. To address whether our proteins behaved as expected based on previous data, we purified 3xFlag fusion full-length proteins from HEK293T cells with Flag M2 resin and subsequently eluted them by a molar excess of 3xFlag peptide. To remove the free peptide, we used centrifugal ultrafiltration with a 100 kDa cutoff membrane. Figure 2A shows a silver stained SDS-PAGE of LRRK1 and LRRK2 purifications after peptide removal by centrifugal ultrafiltration. The estimated purity by densitometric analysis is >90%. Where possible, we used peptide-free proteins for the subsequent characterization described below. To verify that purified proteins are properly folded, we used circular dichroism (CD) spectroscopy and intrinsic fluorescence. CD analysis of LRRK1 and LRRK2 wild-type shows that both proteins are folded with similar content of predicted secondary structures (Fig. 2B). Protein concentration was calculated by densitometry of protein stained gels using BSA standards (Fig. S1). Specifically, LRRK1 possesses about 79% of alpha helices, 14% of beta sheets and 7% of random coils, whereas LRRK2 displays 75% content of alpha-helix, 15% of beta sheet and 10% of random coils. As shown in figure 1B, the two GTP-deficient binding mutants, namely LRRK1-K650A and LRRK2-T1348N, consistently display lower steady state levels. One possibility is that lack of GTP binding prevents the nucleotide-binding pocket to fold correctly, resulting in a partially unfolded protein. To assess this, we compared CD spectra of wild-type vs GTP-deficient binding proteins (Fig. S2). CD analysis did not revealed major conformational perturbations for the GTP-deficient binding mutants, although it is important to point that CD is not the most suitable technique to investigate small changes in secondary structure. Small folding variations could account for the observed reduced stability of these mutants.

Next, given the presence of 31 and 26 tryptophan residues in LRRK1 and LRRK2 respectively, we exploited the intrinsic fluorescence properties of this amino acid to assess protein folding. We observed a maximum emission peak at 334 nm for LRRK2 and 336 nm for LRRK1, which are typical values of folded proteins. Upon addition of 6M guanidinium chloride (GdHCl), a concentration at which any protein becomes randomly coiled, both samples exhibited a red-shifted emission peak at about 355 nm typical of unfolded proteins with increased exposure of the tryptophan to aqueous environment (Fig. 2C). Overall, these results suggest that purified LRRK1 and LRRK2 are folded and appear well behaved in solution.

### LRRK1 and LRRK2 bind guanine nucleotides with similar affinities

**Figure 3:**
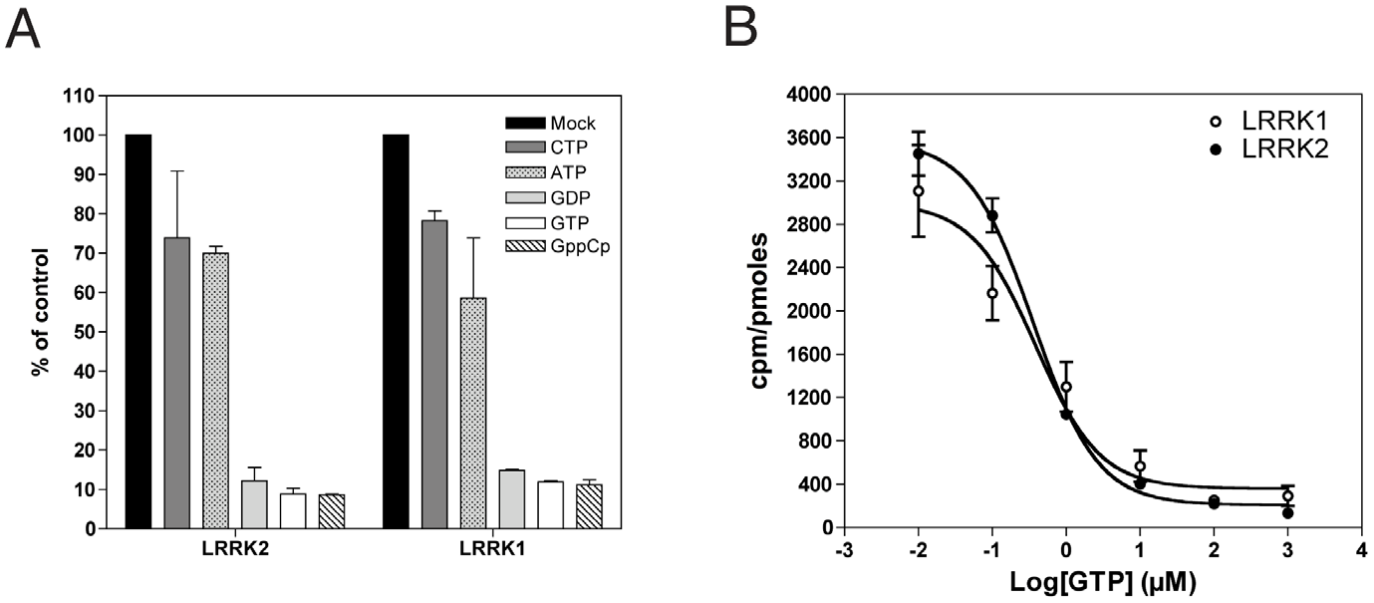
LRRK1 and LRRK2 bind guanine nucleotides. (A) Nucleotide competition assays with purified 3xFLAG-LRRK1/2 bound to M2-Flag affinity resin and incubated with a fixed concentration of GTP-α-P^33^ (10 nM) in the presence of 100 µM of cold nucleotides. Graph shows that loaded GTP- α-P^33^ is outcompeted by guanine nucleotides but not by ATP or CTP. (B) Nucleotide competition assays with proteins incubated with a fixed concentration of GTP- α-P^33^ (10 nM) and varying concentrations of cold GTP. Competition curves with GTP were used to generate IC50 values, which are apparent dissociation constants.

We next addressed whether these apparently folded proteins were able to bind GTP and whether they had kinase activity. Equal amounts of purified 3xFlag-LRRK1/2 bound to M2-Flag affinity resin were suspended in loading buffer and incubated with a fixed concentration of GTP-α-^33^P (10 nM) in the presence of 100 µM of cold nucleotides. As shown in figure 3A, loaded GTP-α-^33^P is outcompeted by guanine nucleotides but not by ATP or CTP. We next investigated the affinity of LRRK1 and LRRK2 for GTP. Again, equal amounts of proteins were incubated with a fixed concentration of GTP-α-P^33^ (10 nM) in the presence of increasing concentrations of non-radiolabeled GTP. Competition curves with GTP were used to estimate apparent dissociation constants (KD_(app)_), corresponding to the GTP concentration inducing 50% of signal loss. Apparent KD_(app)_ were 0.38±0.22 µM and 0.36±0.08 µM for LRRK1 and LRRK2 respectively, suggesting reasonable and similar folding of the GTP binding regions for both proteins (Fig. 3B). ROC-COR domains of LRRK1 and LRRK2 display 26% identity and 46% similarity at the amino acid level, which might explain their rather similar catalytic constants (Fig. S4).

### Kinase activity of LRRK1 versus LRRK2

**Figure 4:**
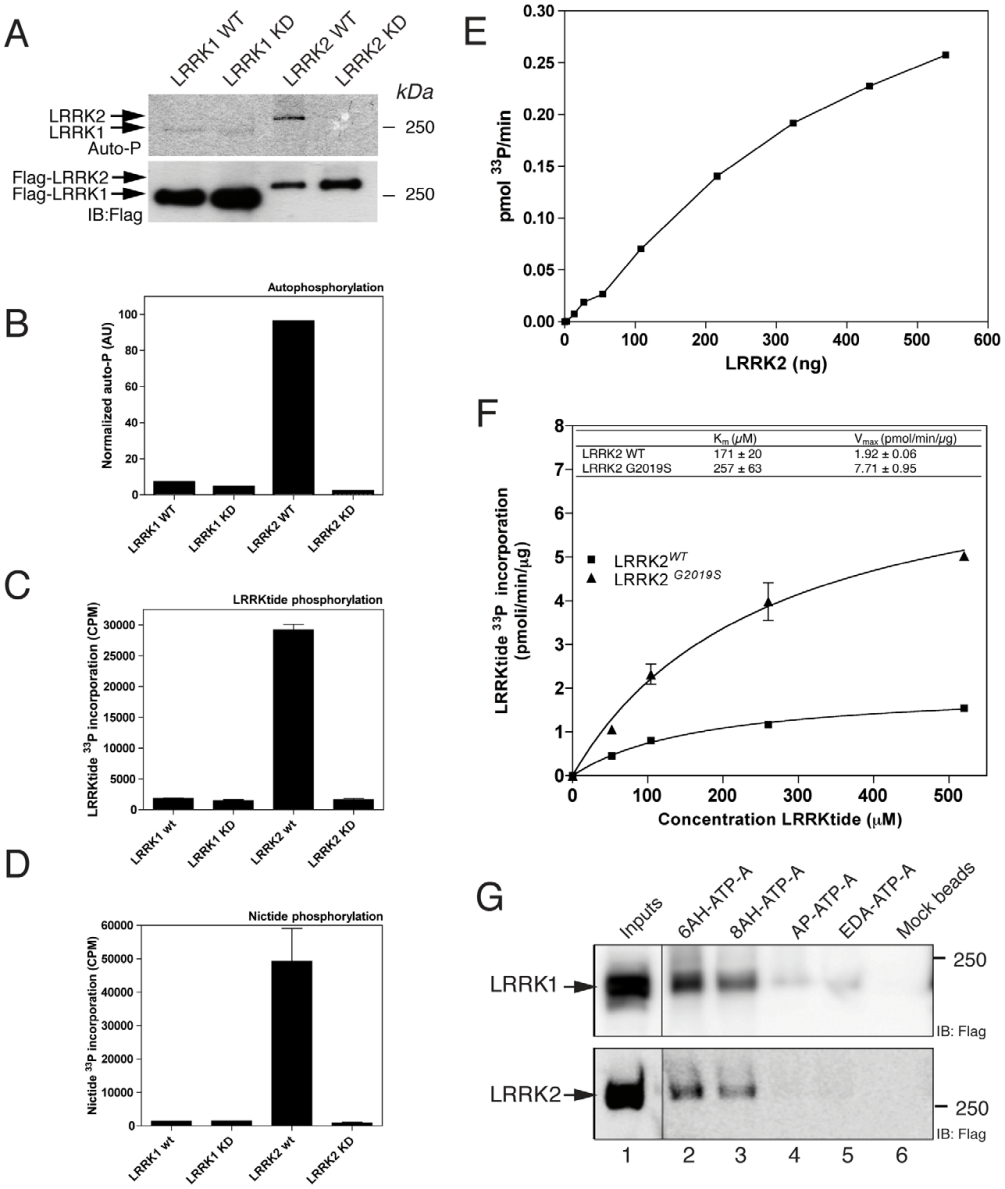
Comparative kinase activities of LRRK1 and LRRK2. (A) Autophosphorylation assays of 3xFlag-LRRK1 wild-type, LRRK1 kinase dead, LRRK2 wild-type and LRRK2 kinase dead. End-point reactions (60 minutes) were resolved on a 4–20% SDS-PAGE and transferred onto PVDF membranes. Upper panel is autoradiography and lower panel western blot to correct activity for total loading (with anti-Flag antibody). The experiment is representative of n = 3 replicates. Markers to the right of the blots are in kilodaltons. (B) Quantitation of ^33^P signal by densitometry normalized to total loading. (C–D) LRRKtide (C) and (D) Nictide phosphorylation assessed by P81 filter binding assay reveals that both peptides are specific substrates for LRRK2. (E) Rate of P^33^ incorporation as a function of LRRK2 protein content (from 10 to 550 ng) measured by LRRKtide phosphorylation assays. (F) Kinetic constants of wild-type and G2019S LRRK2 for LRRKtide were determined by incubating 25 nM LRRK2 with varying concentrations of LRRKtide in the presence of 100 µM ATP and by fitting the data to a hyperbolic function. K_m_ was 171±20 µM for wild-type and 257±63 µM for G2019S. V_max_ were 1.92±0.06 pmol/min/µg for wild-type and 7.71±0.95 pmol/min/µg for G2019S. (G) ATP binding was tested for both LRRK1 and LRRK2 by affinity binding of the proteins to 4 different forms of ATP-bound agarose beads (ie ATP is coupled to the beads in different conformations) as described in materials and methods. Both LRRK1 and LRRK2 bound to the beads when ATP is coupled via the adenine moiety (6-AH-ATP-A or 8-AH-ATP-A). Binding was negligible for ATP coupled via the gamma-phosphate (AP-ATP-A) or ribose group (EDA-ATP-A). The position of the 250 kilodalton M_w_ marker is shown.

The kinase domains of LRRK1 and LRRK2 are relatively conserved with 30% identity and 50% similarity in their aminoacid sequence (Fig. S5). To investigate kinase activity and address substrate specificity, we used recombinant wild-type and kinase dead (KD) proteins purified as described above. LRRK2 shows robust autophosphorylation activity, while LRRK2 KD has only minimal activity. LRRK1 wild-type shows minimal autophosphorylation activity compared to its KD control (Figure 4A–B).

We next asked whether *in vitro* LRRK2 model substrates are also substrates for LRRK1. Therefore we compared LRRK1 and LRRK2 for their ability to phosphorylate LRRKtide, a peptide (RLGRDKYKTLRQIRQ) derived from human moesin [52] and Nictide (RLGWWRFYTLRRARQGNTKQR), derived from LRRKtide [53]. We observed that only LRRK2 wild-type could efficiently phosphorylate both peptides while LRRK1 was unable to phosphorylate LRRKtide or Nictide (Fig. 4C–D). These results confirm previously published data [55].

We further studied the catalytic properties of 3xFlag LRRK2. Specific activities of wild-type and hyperactive mutant G2019S LRRK2 were respectively 2.0 nmol/min/mg and 6.7 nmol/min/mg, consistent with previous reports showing G2019S about 3-fold more active than wild-type [19]. Interestingly, specific activities of 3xFlag-LRRK2^1-2527^ are lower than those reported with truncated GST-LRRK2^970-2527^ [51], confirming previous studies reporting an inhibitory role on kinase activity by the N-terminal region of LRRK2 [54].

We calculated kinetic constants of LRRK2 wild-type and the hyperactive G2019S mutant for LRRKtide. LRRK2 proteins (25 nM) were incubated with varying amount of LRRKtide and 100 µM ATP for 30 minutes. K_m_ were calculated to be 171±20 µM and 257±63 µM and V_max_ were determined to be 1.92±0.06 and 7.71±0.95 pmol/min/µg for LRRK2 wild-type and G2019S respectively (Fig. 4E–F).

### ATP binding properties

Since we could not assess whether the purified LRRK1 was kinase active due to the lack of validated LRRK1 model substrates, we measured the ability of LRRK1 to bind ATP and compared it to LRRK2. As shown in figure 4G, we confirm binding of both proteins to ATP using different ATP agarose beads. Binding to the beads was inhibited by the addition of free ATP (1 mM) to the binding reaction but not GTP (1 mM) (Fig. S6), indicating the specificity of the binding. To further compare the properties of the LRRK1 and LRRK2 kinase domains, we performed sequence homology (Fig. S5A) and comparative homology modeling (for a recent model of LRRK2 kinase, refer to [56]). Based on homology modeling, LRRK1 and LRRK2 kinase display a structural organization of a typical protein kinase: an N-terminal lobe consisting of a five-stranded β-sheet and one α-helix, connected by a hinge region to a predominantly helical C-terminal lobe [57], [58]. The ATP-binding groove lies at the interface of these two lobes. The residues important for ATP-binding and kinase activity are conserved between LRRK1 and LRRK2 with the exception of the aromatic amino acid, which normally shields the site for phosphate transfer, in the P-loop of LRRK1. However, one important difference between both kinases is the presence of an extra loop between β-sheet 5 and α-helix C1 of the N-terminal lobe (Fig. S5B). As shown in figure 4G, this difference does not inhibit the binding of ATP to LRRK1, however this insertion in LRRK1 kinase may affect substrate binding and may therefore explain the divergent kinase data.

Based on the interpretation of these results suggesting these are correctly folded proteins, we next probed the structure of highly purified LRRK1 and LRRK2.

### Analysis of particle size and distribution by transmission electron microscopy (TEM)

We imaged purified LRRK1 and LRRK2 by TEM. The two wild-type proteins were diluted to 5 ng/µl, negatively stained with uranyl acetate and either directly observed at the microscope or further labeled with primary M2 anti-Flag antibodies and secondary antibodies conjugated with 5 nm gold particles. For both proteins, we observed that the majority of the particles were similar in size with a minor proportion being more heterogeneous. Large (>400 Å), heterogeneous and amorphous aggregates were excluded from the analysis as they did not represent a homogeneous population of particles.

**Figure 5:**
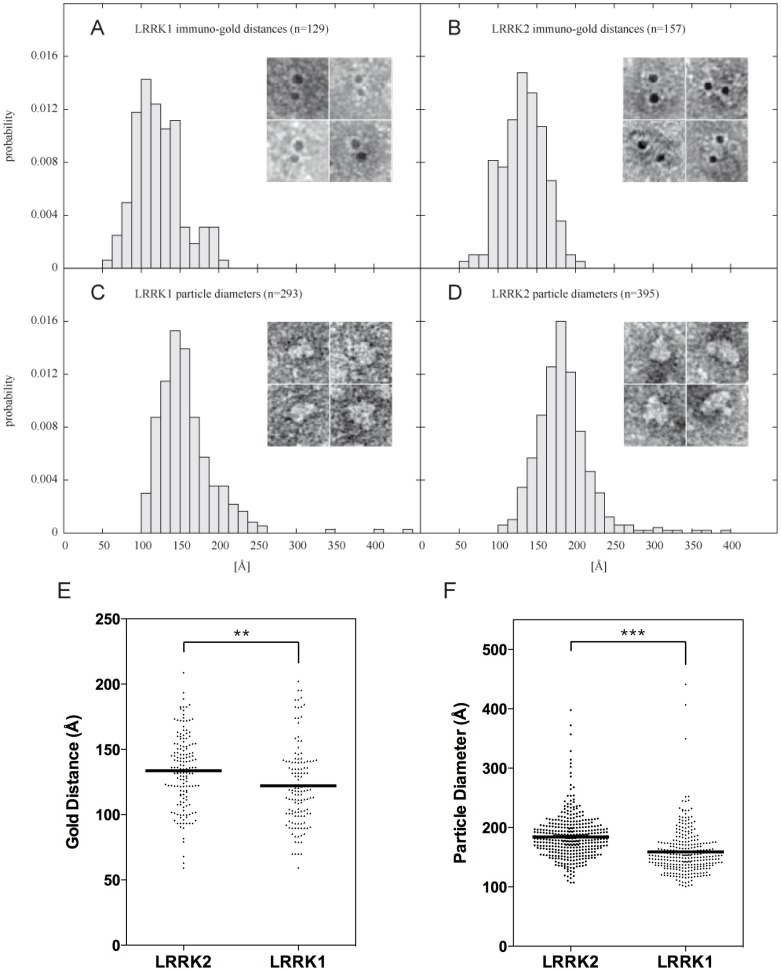
Analysis of LRRK1 and LRRK2 structure by transmission electron microscopy (TEM). Distributions of gold particle distances of double-gold labeled LRRK1 (A) and LRRK2 (B) particles and representative images. Particles were stained with uranyl acetate and subsequently labeled with primary anti-Flag (M2) antibody and secondary 5 nm gold-labeled secondary anti-mouse antibody. (C and D) Distributions of particles diameters of purified LRRK1 and LRRK2 negative stained with uranyl acetate and representative images of protein shapes. (E) Scatter plots of gold distances measured for double-gold labeled LRRK1 and LRRK2. Distributions are significantly different as assessed by t-test (**, *P* = 0.001). (F) Scatter plots of particle size for LRRK1 and LRRK2. Distribution of LRRK1 and LRRK2 are significantly different by t-test (***, *P*<0.0001).

Immunogold labeling of the purified preparations revealed the presence of three groups of particles: a large number of particles were not labeled at all, about 20% were labeled with one gold particle and about 5% were double-labeled. Antibodies pre-absorbed with 3xFlag peptide showed only background signal, confirming specificity of the labeling (Fig. S7). We selected 129 and 157 double gold stained particles for LRRK1 and LRRK2 respectively from three independent purifications. Gold particle distributions (Fig. 5A–B) are centered around 100 Å and 130 Å for LRRK1 and LRRK2 respectively.

Double-labeled particles are likely to represent dimeric LRRK1 and LRRK2. Single-labeled particles may either represent monomers or, alternatively, dimers with only one epitope labeled. The latter interpretation is supported by two further pieces of evidence. First, only about 20% of particles were labeled, indicating that labeling efficiency is not 100% (as expected) and, as a consequence, the predicted frequency of double-labeled molecules is the product of the probability of a single labeling event (0.20×0.20 = 0.004, i.e. 4%) which is close to the experimental 5% observed. To further address whether double-labeled particles could be dimeric LRRKs, we compared these distances with the mean size of the particles and imaged 293 and 395 negative stained particles for LRRK1 and LRRK2 respectively from three independent purifications (Fig. 5C–D). For both proteins, we observed only one population of particles with a mean diameter of 145 Å for LRRK1 and 175 Å for LRRK2. The asymmetry of the Gaussian distributions may indicate that particles are elongated in shape. The distance of two gold particles is significantly different (P = 0.001) between LRRK1 and LRRK2 (Fig. 5E) as are the particle diameters (P<0.0001) (Fig. 5F). The fact that the distribution of distances between two gold particles is very narrow suggests that the particles analyzed are ordered dimers, in which the two Flag epitopes conserve their relative orientation in the protein particle.

To further support the formation of dimeric LRRK proteins, we performed immunogold staining of purified LRRK1 and LRRK2 before and after addition of 6M GdHCl. As shown in figure 2C, 6M GdHCl induces protein unfolding and is therefore predicted to alter the dimeric conformation. We imaged 20 random fields at TEM and measured all possible distances among particles in order to obtain an unbiased dataset of measurements and normalized each group of distances (bin size 2.5 µm) by annulus area (Fig. S3). The distribution of distances in non-denatured samples displays a peak at 100–200 Å, which corresponds to dimeric LRRKs (Fig. S8). Addition of 6M GdHCl causes a random distribution of distances with loss of the peak corresponding to dimeric LRRKs (Fig. S8).

**Figure 6:**
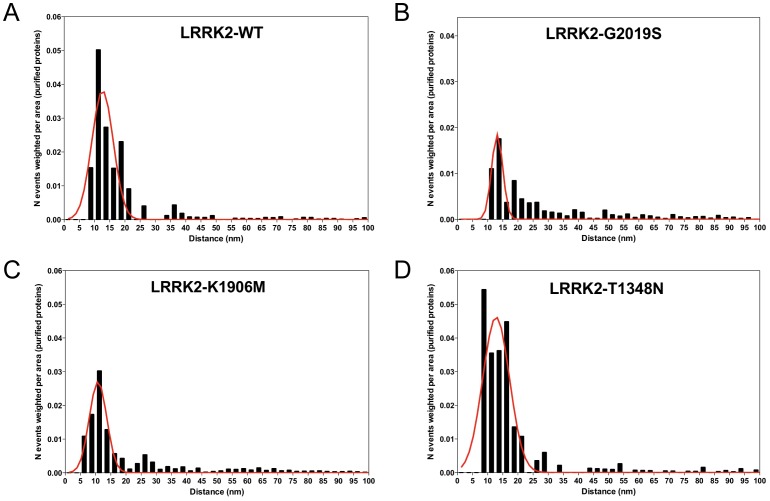
Analysis of different LRRK2 variants by immunogold EM reveals existence of dimeric proteins. Distributions of gold particle distances of gold-labeled LRRK2 wild-type (A), G2019S pathological mutant (B), kinase dead K1906M (C) and GTP deficient binding mutant T1348N (D). Distances between particles were measured within 200 nm and weighted by the area of the annulus of thickness correspondent to the bin size (2.5 nm). See the materials and methods section for a more detailed explanation of the analysis.

Next, we assessed the presence of dimeric LRRKs for wild-type, artificial and pathological mutants by immunogold EM. Again, we calculated all possible distances among particles to obtain an unbiased dataset of measurements and normalized each group of distances (bin size 2.5 µm) by annulus area (Fig. S3). As shown in figure 6, all LRRK2 variants analyzed retain the ability to dimerize, although there is variation in the fraction of dimers. Specifically, the pathological mutant G2019S and the kinase dead K1906M displayed a reduced number of dimeric molecules (Fig. 6B–C) compared to wild-type and T1348N (Fig. 6A and D). LRRK1 kinase dead (K1269W) and GTP deficient binding (K650A) mutants also retain the ability of forming dimers, although LRRK1-K1269W samples displayed reduced double-labeled particles (Fig. S9). However, given that immunogold labeling is an indirect assessment of quaternary structure which depends on the epitope accessibility to the antibody, we cannot perform cross-comparisons of the relative proportions between dimers vs other conformers or speculate that pathological mutations affect the fraction of dimerized protein, but only conclude that mutations do not disrupt the ability of forming dimers.

Overall, our data demonstrate that highly purified LRRK2 and its closest homolog LRRK1 form dimers.

### Endogenous LRRK2 visualized by TEM from cell lysates

**Figure 7:**
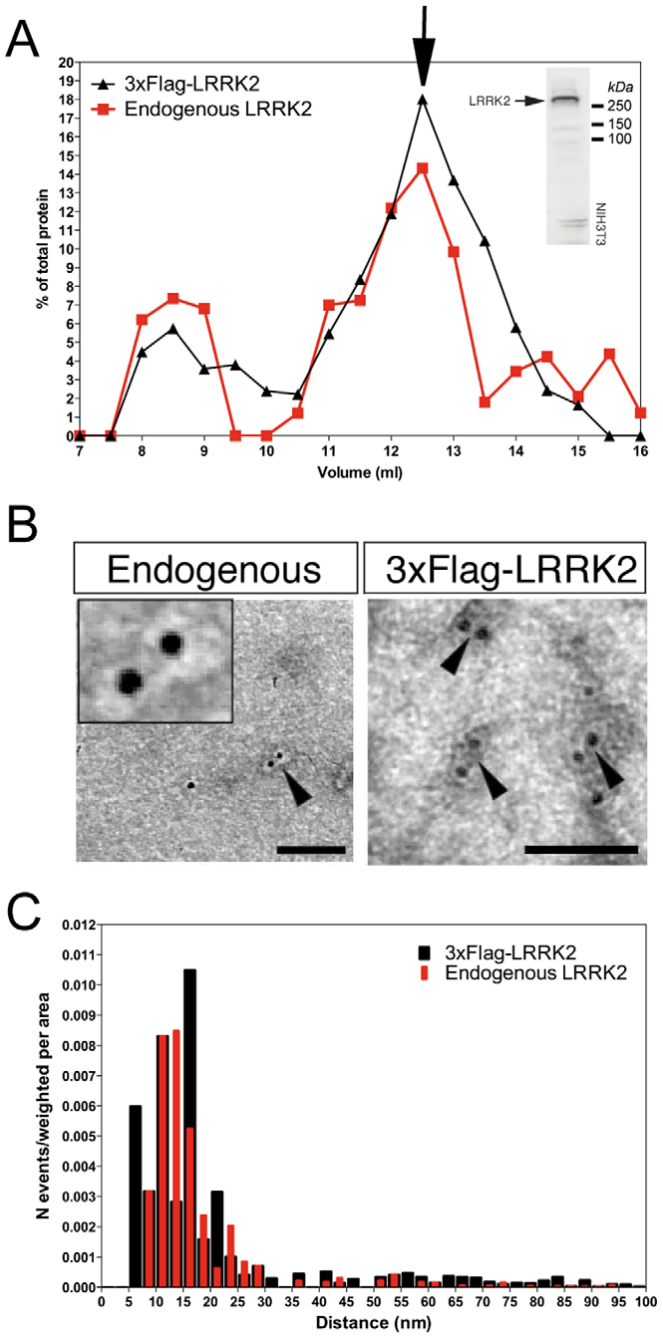
Immunogold labeling of endogenous and 3xFlag-tagged ectopic LRRK2 chromatographic fractions from cell lysates. (A) Relative chromatographic profiles built by measuring the dot blot immunoreactive signal intensity of each fraction and dividing it by the total signal of the protein in all fractions to determine the percentages shown in the graph. Endogenous LRRK2 from NIH-3T3 cells was detected using a rat monoclonal antibody [68], which recognizes endogenous LRRK2 as shown by western blot of a total lysate (inset image). (B) Representative TEM images of immunogold labeled samples from chromatographic fractions corresponding to 12.5 mL peaks. 3xFlag-LRRK2 was labeled using monoclonal M2 anti-flag antibodies and anti-mouse secondary antibodies conjugated with 5 nm gold particles; endogenous LRRK2 from NIH-3T3 cells was labeled using a anti-LRRK2 rabbit monoclonal antibody (aa 100–500) and anti-rabbit secondary antibodies conjugated with 10 nm gold particles. (C) Frequency distribution plots of gold-particle distances for 3xFlag-LRRK2 (black bins) and endogenous (red bins) chromatographic fractions analyzed by immunogold EM.

We took advantage of immunogold labeling to visualize endogenous LRRK2 from size exclusion chromatography (SEC) fractionated lysates of LRRK2 stable HEK293T lines or NIH3T3 cells with endogenous LRRK2 expression. As shown in figure 7A, both endogenous and exogenous LRRK2 are mainly present in the 12–13 mL fraction corresponding to an apparent molecular weight of 600 kDa according to the elution volumes of calibration molecules (Fig. S10). This is consistent with previous findings [8], [25], [26], [59]. Of interest, well-characterized LRRK2 partners such as 14-3-3, beta-tubulin and Hsp90 cannot be detected in the 600 kDa fraction under equivalent experimental conditions used for detection from total lysates of NIH3T3 (Fig. S11). However, it is possible that these proteins co-fractionate with LRRK2 but their concentration is below the detection limit of the experimental set up as we could observe co-fractionation of endogenous 14-3-3 with overexpressed LRRK2 (data not shown). The 12–13 mL fractions were deposited on carbon-coated grids and immunogold labeled with primary anti-Flag or rabbit monoclonal anti-LRRK2 (aa-100–500), which has been used successfully in a non-denaturing application, namely immunoprecipitation [7]. We observed single but also double-labeled gold particles in both endogenous and exogenous samples (Fig. 7B), similarly to purified proteins. Analysis of reciprocal distances among particles (performed as previously described) revealed the presence of one single distribution centered at 100–150 Å for both endogenous and 3xFlag proteins (Fig. 7C). These distributions are consistent with those obtained using purified proteins (Fig. 5 and 6).

## Discussion

In this study we investigated for the first time the quaternary structure of full-length LRRK2 and of its closest homolog LRRK1 by transmission electron microscopy. A major hurdle to this type of analysis has been the obtainment of pure full-length LRRK2 in sufficient quantities suitable for structural characterization. Several attempts have been made to obtain LRRK2 fragments from prokaryotic sources [60], [61], [62], however the only proteins reliably demonstrating kinase activity have been purified from eukaryotic cells as truncated proteins [51], [52]. Here, we developed a protocol to purify full-length recombinant soluble LRRK1 and LRRK2 proteins from mammalian cells by immunoaffinity chromatography. We used a combination of spectroscopic measurements and enzymatic assays to demonstrate that LRRK1 and LRRK2 retained native folding and enzymatic activities and were therefore suitable for single particle analysis by transmission electron microscopy using negative staining and immunogold labeling.

The regions of highest homology between LRRK1 and LRRK2 are in the LRR domain and the catalytic ROC-COR-kinase tridomain. Our results suggest that sequence similarity is reflected in functional similarity for some domains. GTP binding affinities of LRRK1 and LRRK2 were comparable using an isotopic displacement assay and are in the same range as previously reported for LRRK2 using a similar assay [21]. The only other ROCO protein for which GTP binding affinities have been measured is the *C. tepidum* ROCO for which a similar GTP dissociation constant was reported (0.5 µM for GppNHp, 13.4 µM for GDP) [32].

While both LRRK proteins can bind to ATP as predicted for kinases, their kinase activity revealed striking differences. In particular we could not observe LRRK1 kinase activity toward itself or against generic or LRRK2 specific substrates, an observation that may be, in part, explained by the presence of an extra loop between β-sheet 5 and α-helix C1 of the N-terminal lobe. This loop possibly hinders substrate binding. Methods to assess LRRK2 phosphorylation have been well characterized and include autophosphorylation [63], [64], [65] or the phosphorylation of LRRK2 specific peptides such as LRRKtide or Nictide [52], [53]. In all cases here, the phosphorylation activity of LRRK1 was below detection limits for substrate phosphorylation assays and very low for autophosphorylation activity. These results are in agreement with another recent study where the authors did not observe significant autophosphorylation activity for LRRK1 [55]. It is interesting to note that in a previous study where we assessed LRRK1 and LRRK2 autophosphorylation activity using proteins bound to affinity resins [66], we observe only small differences in autophosphorylation activity, indicating the importance of working with soluble and pure protein preparations to improve the signal to noise ratio. We conclude that LRRK1 phosphorylation properties are fundamentally different from those of LRRK2, substrate specificity differs and while autophosphorylation is robust in LRRK2, it is not likely to play a significant role for LRRK1. Therefore, although the kinase domains are similar for LRRK1 and LRRK2, at a functional level the two proteins are likely to be very different.

To explore whether LRRK1 and LRRK2 are dimeric, we used these biochemically validated proteins to investigate their quaternary structure. Although crystallography represents the ideal approach to gain insight into the details of a protein structure, transmission electron microscopy (TEM) has the unique advantage of requiring diluted samples for image analysis and is ideal for molecules larger than 300 kDa such as LRRK1 and LRRK2. Several lines of evidence support the notion that active LRRK2 is dimeric [24], [27], [51], [67]. However, these studies are based on protein fractionation of cell lysates where predicting protein molecular weight, and by inference monomer vs dimer state, is difficult because of the presence of heterologous binding partners.

Here we show for the first time that highly purified LRRK1 and LRRK2 are capable of forming dimers as revealed by the presence of double-gold labeled LRRK1 and LRRK2 particles imaged by TEM. The distance between two gold particles is smaller than the mean particle size of LRRK2 suggesting that the N-terminal region, which is labeled by anti-Flag antibodies, possesses a well-defined structure. In particular, the width of the half height of the curve that interpolates the distribution of distances between gold particles is around 60 Å. Considering that the complex of primary and secondary gold-labeled antibodies is oriented randomly, the N-terminal region is likely to possess a structure that places the epitopes in a defined position. For LRRK1, the width of the half height of the curve is approximately 100 Å suggesting that the N-terminal region is possibly more flexible. Importantly, addition of a denaturing agent such as 6M GdHCl, that induces unfolding of the protein as witnessed by the red-shift of tryptophan fluorescence emission, caused a sharp decrease in the number of double-gold labeled particles compatible with the dimer size, supporting the notion that folded proteins are compact dimers. Collectively, these results support the hypothesis that LRRK proteins are capable of forming dimers in the absence of other binding partners. We also analyzed the effect of point mutations disrupting kinase activity, GTP binding or the hyperactive pathogenic G2019S mutation on dimer formation. Although we observed a reduction in the number of immunogold doublets in the G2019S and kinase dead mutants, it is difficult to conclude that these proteins are less prone to assemble in dimers rather than presenting small structural perturbations, which reflect on the antibody-antigen affinity. Interestingly, purified GTP-dead LRRK2 (T1348N) retains ability to form dimers, indicating that loss of GTP-binding capacity, although likely to perturb the three dimensional structure, is not sufficient to abolish dimerization. This is consistent with previous reports showing that LRRK2 self-interacts via multiple different domains [24].

We have also been able to visualize dimeric LRRK2 particles from chromatographic fractions corresponding to an apparent molecular weight of 600 kDa, which have been probed for endogenous LRRK2. There are now several proteins that have been shown to interact with LRRK2, including 14-3-3, HSP-90 and tubulin and whether these contribute to the ∼600 kDa peak has not been addressed. Given that immunogold EM was performed on enriched protein fractions rather than highly pure sample due to technical limitations on purifying endogenous material, we cannot rule out whether the chromatographic peak contains both dimeric and monomeric LRRK2 with binding partners. A recent paper by Ito and Iwatsubo presents intriguing observations supporting the conclusion that LRRK2 from lysates is predominantly monomeric [41]. As the SEC profiles and immunogold particle distance distributions are comparable in the stable lines compared to endogenous LRRK2 in NIH-3T3 lysates, we can conclude that LRRK2 forms dimers in cells, probably co-existing with monomers.

The next key steps will be to pursue in more detail the structure of LRRK proteins and its links to their biology. The validated LRRK proteins presented here can be used to investigate their conformation by cryo-electron microscopy, in order to obtain more detailed structural information to model their tridimensional structure. The correlation of more detailed structural information of LRRK1 and LRRK2 to their cell biological properties such as how dimerization is linked to cellular complex formation will allow a better understanding of how LRRK biochemical properties impact LRRK mediated cellular functions. This is of particular interest for LRRK2 whose biochemical properties, such as its kinase activity, have been proposed as potential targets for disease modifying Parkinson's disease therapy.

## Methods

### Constructs

Eukaryotic expression constructs of 3xFlag tagged LRRK2 (pCHMWS-3xFlag-LRRK2) were generated as described [16]. LRRK1 sequences were amplified from pCMV-2myc-LRRK1 (a generous gift of Dr. Bertram Weiss [11]) by PCR using forward primer with a BamHI overhang GAA TTC GGA TCC ATG GAG ACG CTT AAC GGT GCC G and reverse primer with SpeI overhang GAA TTC ACT AGT TTA CCT TCT CTT GCG AGT GCA AGC. The PCR amplicon was digested with BamHI and SpeI and cloned into the multiple cloning site of the pCHMWS backbone [42]. Mutant forms of LRRK1 and LRRK2 constructs were generated using the QuickChange mutagenesis kit (Agilent Technologies, CA, USA) according to the manufacturer's instructions.

### Cell culture and transfection

HEK293T cells were cultured in Dulbecco's modified Eagle's medium supplemented with 10% fetal bovine serum at 37°C and 5% CO_2_.

Lentiviral vectors (LVs) encoding human 3xFlag-LRRK1 and 3xFlag-LRRK2 under control of the cytomegalovirus (CMV) promoter were prepared as described previously [43]. For transduction in cell culture, 50,000 HEK293T cells were plated in a 24-well plate and grown in DMEM with 10% fetal calf serum. The next day, vector was applied to the HEK293T cells for 2 days. This procedure was repeated twice with the same cells to obtain triple transduced cells. These cells were then expanded for use in experiments.

### Protein purification

Cells were solubilized in lysis buffer containing 20 mM Tris-HCl pH 7.5, 150 mM NaCl, 1 mM EDTA, 0.5% Tween 20 or 1% Triton X-100, 2.5 mM sodium pyrophosphate, 1 mM beta-glycerophosphate, 1 mM NaVO_4_, protease inhibitor cocktail (Sigma), and lysates were centrifugated for 30 minutes at 14000×g. Afterwards, lysates containing 3xFlag-tagged protein were incubated with anti-Flag M2 agarose beads for 2 hours at 4°C on a rotator. After extensive washing (2 washes with Tris-HCl 20 mM, NaCl 500 mM, Tween 0.5%, 2 washes with Tris-HCl 20 mM, NaCl 300 mM, Tween 0.5%, 2 washes with Tris-HCl 20 mM, NaCl 150 mM, Tween 0.5%, 2 washes with Tris-HCl 20 mM, NaCl 150 mM, Tween 0.1%, 2 washes with Tris-HCl 20 mM, NaCl 150 mM, Tween 0.02%), proteins were eluted in elution buffer (20 mM Tris-HCl pH 7.5, 150 mM NaCl, 0.02% Tween 20 or 0.02% Triton X-100 and 150 ng/µl of 3xFlag peptide) by shaking for 30–40 minutes at 4°C. Excess 3xFlag peptide (Sigma) was exchanged using Vivaspin 6 (Sartorius) or dialysis devices with molecular weight cut off of 100 KDa (Spectrum Laboratories).

### Size Exclusion Chromatography (SEC)

Cells were lysed as above and cleared lysates (0.5 ml) were injected and separated on a Superose 6 10/300 column (GE Healthcare). The column was pre-equilibrated with buffer (20 mM Tris-HCl pH 7.5, 150 mM NaCl and 0.07% Tween 20) and used at a flow rate of 0.5 ml/min. Elution volumes of standards were 7.5 ml for Blue Dextran (V_0_), 11.5 ml for hemocyanin from *Carcinus aestuarii* (900 kDa), 12 ml for thyreoglobin (669 kDa), 14 ml for ferritin (440 kDa).

Fractions (1 or 0.5 ml) were analyzed by dot blot. One microliter of each fraction from SEC was applied onto a nitrocellulose membrane. The membrane was blocked with 10% (w/v) milk in TBS plus 0.1% Triton (TBS-T) for 1 hour and subsequently incubated with mouse monoclonal anti-Flag M2-peroxidase (Sigma). Immunoreactive proteins were visualized using enhanced chemiluminescence plus (ECL plus, GE Healthcare, Little Chalfont, England).

### SDS PAGE and Western immunoblotting

Between 2 and 10 µg of protein per well was resolved on 4–20% Tris-glycine polyacrylamide gels (Biorad) in SDS/Tris-glycine running buffer or on NuPAGE® 3–8% Tris-acetate Gel (Invitrogen). Precision Plus molecular weight markers (Biorad) were used for size estimation.

Resolved proteins were transferred electrophoretically to polyvinylidenedifluoride (PVDF) membranes in cold transfer buffer containing 10% methanol. The PVDF sheets were blocked in TBS-T plus 5% nonfat dry milk for 1 hour at 4°C and then incubated overnight at 4°C with ant-Flag-M2 antibody in PBS-T plus 5% non-fat dry milk. The PVDF membranes were washed in TBS-T (3×10 min) at room temperature (RT) followed by incubation for 1 h at RT with horseradish peroxidase-conjugated anti-mouse IgG. The blots were washed in TBS-T (4×10 min) at RT and rinsed in TBS, and immunoreactive proteins were visualized using enhanced chemiluminescence plus (ECL+, GE Healthcare, Little Chalfont, England).

### Circular Dichroism (CD)

CD measurements were carried out on a JASCO J-715 spectropolarimeter interfaced with a personal computer. The CD spectra were acquired and processed using the J-700 software for Windows. All experiments were carried out at room temperature using an optical path length of 0.1 cm. All spectra were recorded in the wavelength range of 198–260 nm, using a bandwidth of 2 nm and a time constant of 8 s at a scan speed of 50 nm/min. The signal to noise ratio was improved by accumulating four scans. Spectra were acquired using 80 nM proteins in 20 mM Tris/HCl buffer (pH 7.5), 150 mM NaCl and 0.02% Tween 20. All spectra are reported in terms of mean residue molar ellipticity (deg cm^2^ dmol^−1^). Protein concentration was determined by using a calibration curve generated with known amounts of bovine serum albumin (BSA) (Fig. S1).

### Intrinsic fluorescence

Fluorescence emission spectra were recorded on a Cary Eclipse fluorescence spectrophotometer (Varian, Agilent Technologies, Santa Clara, CA) using the Cary Eclipse program. Sample measurements were carried out using optical path length of 10 mm. Fluorescence spectra were obtained using an excitation wavelength of 288 nm, with an excitation bandwidth of 5 nm. Emission spectra were recorded between 300–400 nm at a scan rate of 30 nm/sec. Spectra were acquired using 80 nM proteins in 20 mM Tris/HCl buffer (pH 7.5), 150 mM NaCl and 0.02% Tween 20.

### Radiometric assays

For autophosphorylation experiments, proteins (50 nM) were incubated with 100 µM ^33^P-ATP (1 µCi) in kinase reaction buffer consisting of 25 mM Tris-HCl (pH 7.5), 5 mM beta-glycerophosphate, 2 mM dithiothreitol (DTT), 0.1 mM Na_3_VO_4_, 10 mM MgCl_2_ at 30°C for 1 hour min in a final reaction volume of 25 µl; reactions were terminated by the addition of 1 mM EDTA. Autophosphorylation was detected by running samples on 4–12% SDS-PAGE gels and transferring to PVDF membranes. Incorporated ^33^P-ATP was detected by autoradiography and the same membranes were probed with anti-Flag antibody for total protein loading.

For LRRKtide and Nictide ^33^P incorporation, reactions were set up as above but with the addition of 400 µM peptide. The specific activity for LRRKtide of each protein preparation was determined by spotting the reaction mixture to P81 phosphocellulose paper after 0, 5, 15 and 30 minutes. ^33^P incorporation into LRRKtide was quantified by washing the phosphocellulose membranes in 50–75 mM phosphoric acid and liquid scintillation counting.

To determine the kinetic parameters of LRRK2 using LRRKtide as substrate, the assay was performed with varying concentration of peptide. K_m_ and V_max_ values were calculated with GraphPad Prism using non-linear regression analysis.

### Isotopic nucleotide binding assay

Nucleotide binding was performed as described [44], with some modifications. Briefly, anti-Flag agarose beads containing equal amounts of 3xFlag-LRRK1 and 3xFlag-LRRK2 as calculated by densitometry using a standard curve with bovine serum albumin (BSA), were rinsed in loading buffer (25 mM Tris-HCl pH 7.5, 150 mM NaCl, 5 mM EDTA and 0.02% Triton X-100) and incubated with GTP-α-^33^P alone or with 200 µM CTP, ATP, GDP or non-hydrolysable GppCp for 30 minutes with gentle shaking. After incubation, excess nucleotide was removed by rinsing beads three times in kinase buffer. The amount of bound isotopic GTP was measured by scintillation counting.

Aliquots of 3xFlag-LRRK1 and 3xFlag-LRRK2 on agarose beads were preincubated in the presence of GTP-α-^33^P (10 nM) and then equilibrated with different amounts of non-radiolabeled GTP (100 nM–1 mM in tenfold concentration increments).

### Binding to ATP-sepharose beads

ATP binding of LRRK1 and LRRK2 was assessed by pulldown from cell lysates with four different types of ATP-agarose (Aminophenyl-ATP-Agarose, C10-spacer (AP-ATP-Agarose), 8-[(6-Amino)hexyl]-amino-ATP-Agarose (8-AH-ATP-Agarose), N6-(6-Amino)hexyl-ATP-Agarose (6-AH-ATPAgarose), 2′/3′-EDA-ATP-Agarose (EDA-ATPAgarose), according to manufacturer's instructions (Jena Bioscience, Jena, Germany). In brief, HEK293T cells with stable expression of 3xFlag-LRRK1 and 3xFlag-LRRK2 were lysed in ice cold lysis buffer B (Tris 25 mM pH 7.4, NaCl 150 mM, MgCl2 5 mM, DTT 1 mM, NP-40 0,2%, glycerol 10%) containing protease and phosphatase inhibitor cocktails (Roche Applied Science, Vilvoorde, Belgium) and cleared by centrifugation for 10 minutes at 14000×g and 4°C. Cleared lysates were depleted of ATP by triple dialysis against lysis buffer B using dialysis membranes with a molecular weight cutoff of 6–8 kDa (Spectrum laboratories, Breda, The Netherlands) and with a sample-to-dialysis-buffer volume ratio of at least 1∶100 per dialysis step. 200 µg lysate was used per binding reaction to the 4 abovementioned forms of ATP-agarose, as well as for empty agarose beads (negative control) or gamma-aminohexyl-GTP-agarose (Jena) as a positive control. As additional negative controls, binding reactions were also performed in the presence of excess nucleotide (1 mM ATP or GTP). Binding was performed by end-over-end mixing for 1 hour at 4°C after which beads were washed 4 times in lysis buffer B. Proteins were eluted using 2× SDS loading buffer B (Tris-HCl 160 mM pH 6.8, SDS 2%, DTT 0.2 M, glycerol 40%, bromophenol blue 2 mg/ml) and analyzed via western blot as described above.

### Electron microscopy analysis and gold-labeling

3x-Flag-LRRK1/2 purified proteins or endogenous LRRK2 enriched fractions were examined by electron microscopy (EM) followed by immunogold analysis. LRRK-enriched fractions were diluted 20 times in lysis buffer (LB) and purified proteins were diluted to 5 ng/µl in EB (20 mM Tris-HCl pH 7.5, 150 mM NaCl, 0.02% Tween 20 or 0.02% Triton X-100 and 150 ng/µl of 3xFlag peptide). Negatively stained LRRK1 and LRRK2 samples were prepared using previously described methods [45]. A 30 µl drop of sample solution was adsorbed to a glow-discharged carbon-coated copper grid, washed with two drops of deionized water, and stained with two drops of freshly prepared 1% uranyl acetate. To gold-label, samples were incubated for one hour with mouse monoclonal anti-Flag M2 for 3xFlag-tagged proteins or anti-LRRK2 rabbit monoclonal antibodies [46] for endogenous proteins in NIH-3T3 mouse fibroblasts. After 3 washing steps, samples were incubated with 5 nm gold-labeled anti mouse or 10 nm gold-labeled anti rabbit IgG secondary antibodies (Sigma) for 30 minutes. Proteins were adsorbed onto carbon-coated grid and stained following the same procedure used for the non-labeled particles.

Samples were imaged at room temperature using a Fei Tecnai T12 electron microscope equipped with a LaB6 filament and operated at an acceleration voltage of 100 kV. Images were taken at calibrated magnifications in the range 11,000× to 26,000×. Per each field analyzed (500 nm×500 nm), reciprocal distances among immunogold labeled proteins were obtained using ImageJ (NIH, Bethesda, MD, USA). Distances greater than 200 nm (2000 Å) were not included in the analysis. Frequency distribution of particles distances was carried out using GraphPad Prism (GraphPad Software, La Jolla, CA, USA) setting 2.5 nm as bin size. Because the random probability of finding a second gold particle at a given distance from the first particle increases as a linear function of the distance, we introduced a normalization factor to weigh the probability of each event equally. Each data set was therefore normalized by the annulus area within which the particles were counted (thickness corresponds to the bin size, 2.5 nm):
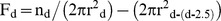
n_d_ represents the number of gold-labeled particles counted inside the annulus area (2 πr^2^_d_)−(2 πr^2^_d-(d-2.5)_) and d is a given distance from the analyzed particles (Fig. S3). Finally, each data set was normalized by the total number of measured distances (about 800 per sample). The distribution of distance is depicted in plots showing the normalized frequencies per inter-immunogold-dot distance.

We also investigated the size of purified LRRK1 and LRRK2 samples without immunogold labeling to optimally visualize particle borders. Size distribution was obtained using the following procedure: particle images were manually extracted from micrographs using the semi-automatic procedure implemented in the BOXER program of EMAN software package [47]. Clearly defined isolated particles were selected and boxed in 60×60 to 150×150 pixel images. Large and amorphous aggregates were manually excluded from the analysis. The particles were subsequently centered and aligned by cross-correlating the individual images to a rotationally averaged image. Once aligned, all images were rotationally averaged to obtain a one dimensional radial intensity profile. Center alignment and rotational averaging were done using SPIDER image processing system [48]. Since in negative stain the particles are bright surrounded by a dark halo of stain, the intensity profiles were fitted by a piecewise function which starts as a constant value (particle intensity) followed by half period cosine drop (simulating the stain halo) and again raising by a half period cosine function until a plateau is reached (constant medium-light background intensity). Particle half-length was taken as a pixel position at the midpoint of the cosine intensity drop. Intensity profile fits and particle size distribution histograms were calculated using MATHEMATICA (Wolfram Research Inc., Champaign, USA).

### Confocal microscopy

Stable HEK293T cells grown into 22 mm coverslips coated with poly-L-lysine were fixed with 4% paraformaldehyde. Cells were stained with primary monoclonal anti-Flag antibodies (clone M2; 1∶200; Sigma). Secondary Alexa 488-conjugated goat anti-mouse antibodies (Molecular Probes) were used to visualize primary antibodies. Images were acquired using a Leica TCS SP5 confocal microscopy.

### Statistical analysis

All quantitative data are expressed as mean ± SEM and represent at least three independent sets of experiments. Significance of differences was assessed by Student's t test.

## Supporting Information

Figure S1 **Protein concentration determination using BSA standards measured by densitometry of silver stained bands.**
(DOCX)

Figure S2 **Circular dichroism analysis of purified 3xFlag-LRRK1 wild-type vs LRRK1-K650A and 3xFlag-LRRK2 wild-type vs LRRK2-T1348N.**
(DOCX)

Figure S3 **Graphical representation of how distance distribution analysis of immunogold stained proteins was conducted.**
(DOCX)

Figure S4 **Alignment of the LRRK2 ROC-COR bi-domain (amino acid 1320 to 1844) with the LRRK1 ROC-COR bi-domain (amino acid 624 to 1207).**
(DOCX)

Figure S5 **Sequence alignment and structure comparison of LRRK1 and LRRK2 KIN domains.**
(DOCX)

Figure S6 **ATP binding of LRRK1 and LRRK2 to ATP is not disrupted in the presence of 1 mM GTP.**
(DOCX)

Figure S7 **Immunogold EM analysis of 3xFlagLRRK2 compared with a pre-adsorbed sample as negative control.**
(DOCX)

Figure S8 **Analysis of frequency distribution of distances between particles in the presence or absence of 6M GdHCl for LRRK1 and LRRK2 wild-type.**
(DOCX)

Figure S9 **Analysis of different LRRK1 variants by immunogold EM reveals existence of dimeric proteins both in cell lysates and purified samples.**
(DOCX)

Figure S10 **Size exclusion chromatography profiles of standard calibration molecules. The table indicates the elution volume of each standard.**
(DOCX)

Figure S11 **Western blot of NIH3T3 total lysate and 12.5 ml chromatographic fraction against known LRRK2 interactors.**
(DOCX)
