# Supplementary material for: Biochemical Characterization of Highly Purified Leucine-Rich Repeat Kinases 1 and 2 Demonstrates Formation of Homodimers
Source: PLoS One. 2012 Aug 29;7(8):e43472. doi: 10.1371/journal.pone.0043472 (PMC3430690; doi:10.1371/journal.pone.0043472)
Supplement: Figure S1 — Protein concentration determination using BSA standards measured by densitometry of silver stained bands. (DOCX) [file pone.0043472.s001.docx]

**Figure S1.**

Protein concentration determination using BSA standards measured by densitometry of silver stained bands.
