# Supplementary material for: Biochemical Characterization of Highly Purified Leucine-Rich Repeat Kinases 1 and 2 Demonstrates Formation of Homodimers
Source: PLoS One. 2012 Aug 29;7(8):e43472. doi: 10.1371/journal.pone.0043472 (PMC3430690; doi:10.1371/journal.pone.0043472)

**Figure S2.**

Circular dichroism analysis of purified 3xFlag-LRRK1 wild-type vs LRRK1-K650A and 3xFlag-LRRK2 wild-type vs LRRK2-T1348N. Representative spectra reported as mean residue molar ellipticity (deg cm^2^ dmol^-1^) of (A) LRRK1 and its GTP-binding deficient form K650A and of (B) LRRK2 and its GTP-binding deficient mutant form T1348N. .


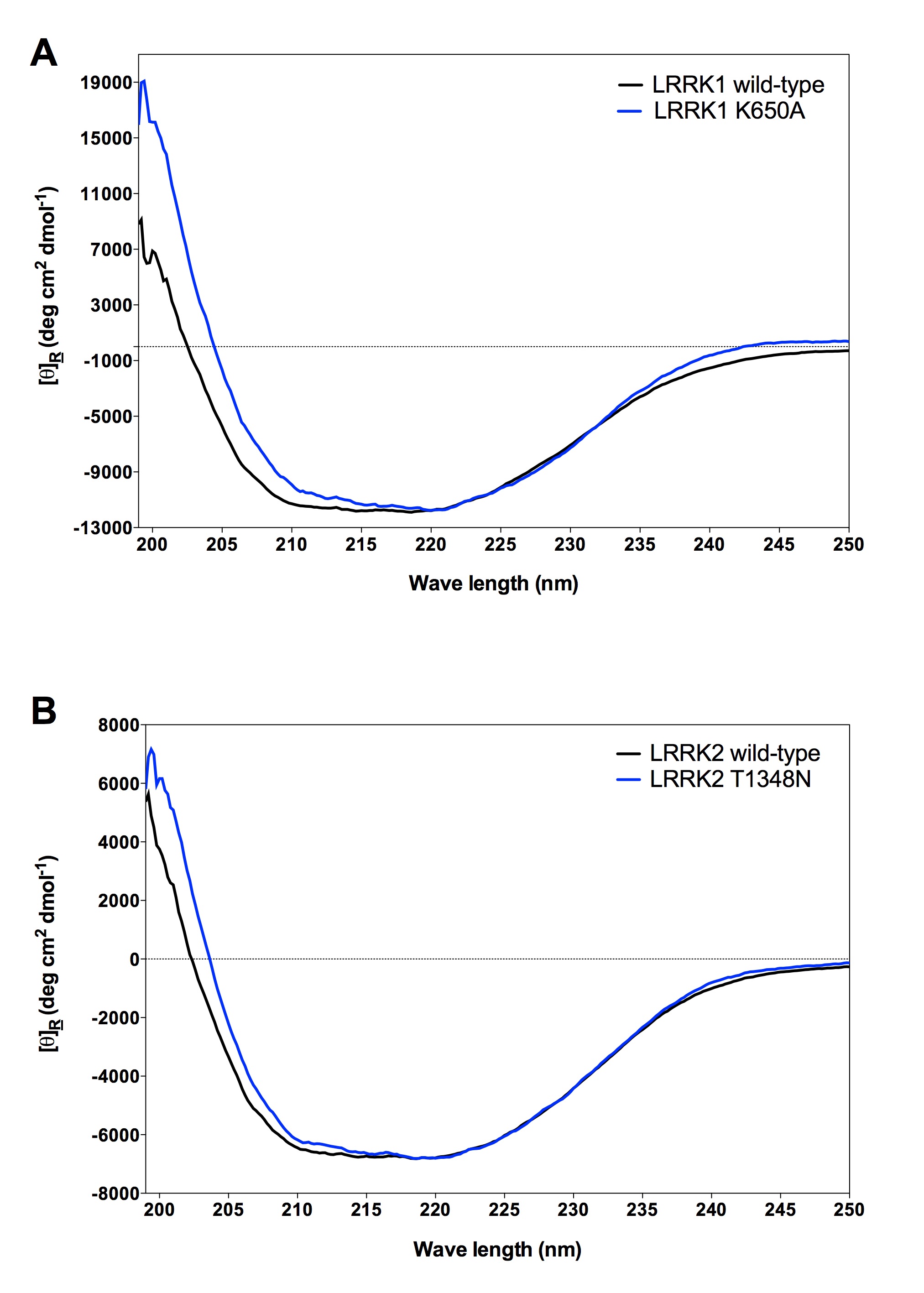

Supplement: Figure S2 — Circular dichroism analysis of purified 3xFlag-LRRK1 wild-type vs LRRK1-K650A and 3xFlag-LRRK2 wild-type vs LRRK2-T1348N. (DOCX) [file pone.0043472.s002.docx]
