# Supplementary material for: Biochemical Characterization of Highly Purified Leucine-Rich Repeat Kinases 1 and 2 Demonstrates Formation of Homodimers
Source: PLoS One. 2012 Aug 29;7(8):e43472. doi: 10.1371/journal.pone.0043472 (PMC3430690; doi:10.1371/journal.pone.0043472)

**Figure S3.**

Graphical representation of how distance distribution analysis of immunogold stained proteins was conducted. From micrographs of 500 nm x 500 nm size, reciprocal distances were automatically measured using Image J (http://rsbweb.nih.gov/ij/). Distances were measured up to 200 nm in order to obtain an unbiased dataset of values. If particles are randomly distributed, we expect that the probability of finding two particles at a certain distance increases with the area of the annulus were a particle (red) falls with respect to the particle in the center of the circle (green). Therefore we assigned a weight to each event (distance) dividing the number of events falling in each bin (2.5 nm) by the area of the corresponding annulus (area of the outer circle minus area of the inner circle).


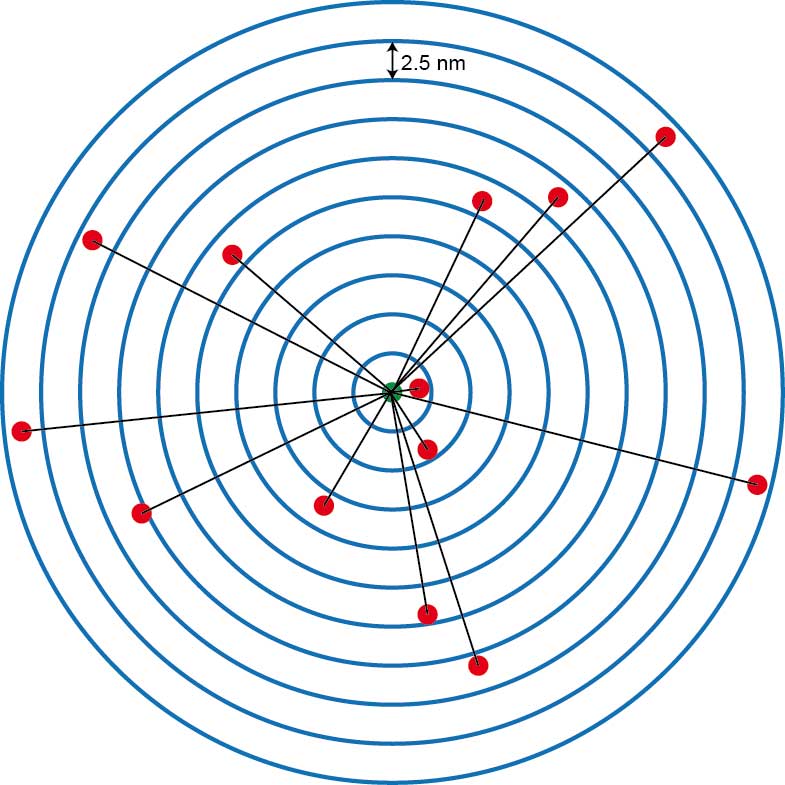

Supplement: Figure S3 — Graphical representation of how distance distribution analysis of immunogold stained proteins was conducted. (DOCX) [file pone.0043472.s003.docx]
