# Supplementary material for: Biochemical Characterization of Highly Purified Leucine-Rich Repeat Kinases 1 and 2 Demonstrates Formation of Homodimers
Source: PLoS One. 2012 Aug 29;7(8):e43472. doi: 10.1371/journal.pone.0043472 (PMC3430690; doi:10.1371/journal.pone.0043472)
Supplement: Figure S4 — Alignment of the LRRK2 ROC-COR bi-domain (amino acid 1320 to 1844) with the LRRK1 ROC-COR bi-domain (amino acid 624 to 1207). (DOCX) [file pone.0043472.s004.docx]

**Figure S4.** Alignment of the LRRK2 ROC-COR bi-domain (amino acid 1320 to 1844) with the LRRK1 ROC-COR bi-domain (amino acid 624 to 1207). Secondary structure elements (NetSurfP [[15](#_ENREF_15)]) are indicated: α-helices are *italic*, and β-strands underlined. Conserved residues are marked with an asterisk (*). LRRK2 pathogenic mutations (R1441C/G and Y1699C), LRRK2 risk factor (R1628P) and LRRK2 functional mutations (K1347A and T1348N) are boxed. Position 1516 (coloured blue), indicates the approximate position transition area between ROC and COR. Putative phosphorylation sites are double underlined. Overall for the ROC-COR tandem domain, identities (marked with an asterisk (*)) are 26% (154/586) (26%); similarities (marked with a plus sign (+)) are 46% (270/586). The identity and similarity scores for the ROC domain are 27% and 48% respectively. For the COR domain, we calculated 25% identity and 44% similarity.

| HsLRRK2_RC  HsLRRK1_RC  HsLRRK2_RC  HsLRRK1_RC  HsLRRK2_RC  HsLRRK1_RC  HsLRRK2_RC  HsLRRK1_RC  HsLRRK2_RC  HsLRRK1_RC  HsLRRK2_RC  HsLRRK1_RC  HsLRRK2_RC  HsLRRK1_RC  HsLRRK2_RC  HsLRRK1_RC  HsLRRK2_RC  HsLRRK1_RC  HsLRRK2_RC  HsLRRK1_RC | 10 20 30 40 50 60  R*FLQQRLK*KAVPYNRMKLMIVGNTGSG*KTTLLQQLMK*TKKSDLGMQSATVGIDVKDWPIQ  S*YLRAQLR*KAEKCKLMKMIIVGPPRQG*KSTLLEILQT*GRAPQVVHGEATIRTTKWELQRP  +*+ +*+** **++*** **+***+ * + + **+ +  70 80 90 100 110 120  IRDKRKRD-LVLNVWDFAGR*EEFYST*HPHFMTQRALYLAVYDLSKGQA*EVDAMKPWLFNI*  AGSRAKVESVEFNVWDIGGP*ASMA*TVNQCFFTDKALYVVVWNLALGEEAVANLQFWLLNI  + * + + **** * + + * * +***+ *++*+ *+ * ++ ** **  130 140 150 160 170 180  *KAR*ASSSPVILVGTHLDVSDEKQRKAC*MSKITKELLNK*R---------GFPAIRDYHFVN  *EAK*APNAVVLVVGTHLDLIEAKFR*VERIATLRAYVLALC*RSPS*GSRATGFPDIT*FKHLHE  +*+* ++ *++******+ + * * ++ + +* *** * *  190 200 210 220 230 240  ATEES-*DALAKLRKTIINESLNFK-----I*RDQLVVGQLIPDCY*VELEKIILSE--R*KNV  ISCKSLEG*QEGLRQLIFHVTCSMKD*VGSTIGCQRLAGRLIPRS*YLSLQEAVLAEQQRRS*R  + +* + **+ * + + + * * * + *+*** *+ *++ +*+* *++  250 260 270 280 290 300  PIEFPVID*RKRLLQLVREN*--QLQLD*ENELPHAVHFLN*ESGVLLHFQDPALQLSDLYFVE  DDDVQYLT*DRQLEQLVEQT*PDNDIKD*YEDLQSAISFLIE*TGTLLHFPDTSHGLRNLYFLD  + + ++* *** + * +* *+ ** *+* **** * + * +***++  310 320 330 340 350 360  *PKWLCKIMAQILT*VKVEGCPKHPKGIIS*RRDVEKFL*SKKRKFP*K*N*YMSQYFKLLEKF*QIA  P*IWLSECLQRIFN*IKGS-RSVAKNGVIR*AEDLRMLL*VGTG-FTQ*QTEEQYFQFLAKF*EIA  * ** + + +* +* *+* *+ * * + ***+ * **+**  370 380 390 400 410 420  LPIGEEYLLVPSSLSDHRPVIELPHCE-NSEIIIRLYEMPYFPM*GFWSRLINRLL-----*  LPVANDSYLLPHLLPSKPGLDTHGMRHPTANTIQRVFKMSFVPVG*FWQRFIARMLISLAE*  **+ + *+* * + + * *+++* + *+*** * * *+*  430 440 450 460 470 480  *-----------------E*ISP*YML*SGRER-------ALRPNRMYWRQGIYLNWSPEAYCL  *MD*LQLFENKKNTKSRNRKVTIYSFTGNQRNRCSTFRVKRNQTIYWQEGLLVTFDGGYLSV  +++ * +* +* * +**++*+ + + +  490 500 510 520 530 540  VGSEVLDNHPES-FLKITVPSCRK*GCILLGQVVDHIDSLMEEWF*PGLLEIDICGE-----  ESSDVNWKKKKSGGMKIVCQSEVR*DFSAMAFITDHVNSLIDQWF*PALTATESDGTPLMEQ  *+* +* +** * + + + **++**+++*** * + *  550 560 570 580  ---GETLLKKWALYS--FNDGEEHQ*K*IL*LDDLMKKA*EEGDLLVNPD  YVPCPVCETAWAQHTDPSEKSEDVQYFD*MEDCVLT*AIERDFISCPR  ** ++ *+ * ++* + * * * + * |
| --- | --- |
