# Supplementary material for: Biochemical Characterization of Highly Purified Leucine-Rich Repeat Kinases 1 and 2 Demonstrates Formation of Homodimers
Source: PLoS One. 2012 Aug 29;7(8):e43472. doi: 10.1371/journal.pone.0043472 (PMC3430690; doi:10.1371/journal.pone.0043472)

**Figure S5.** Sequence alignment and structure comparison of LRRK1 and LRRK2 KIN domains. Sequence alignment of LRRK2 KIN (amino acid 1845 to 2139) with the LRRK1 KIN domain (amino acid 1208 to 1527). Secondary structure elements (NetSurfP [[15](#_ENREF_15)]) are indicated: α-helices are italic, and β-strands underlined. Conserved residues are marked with an asterisk (*). LRRK2 pathogenic (G2019S and I2020T) and functional (K1906M and D1994A) mutations are boxed. Putative phosphorylation sites are double underlined. Identities (marked with an asterisk (*)) = 101/328 (30%); similarities (marked with a plus sign (+)) = 165/328 (50%). In panel B (left), a homology model of LRRK2 kinase (residues 1859 – 2138) is given. The P-loop is shown in red, the conserved K in β3 and E in αC in green, the catalytic loop in purple, the activation loop in yellow, the gatekeeper (gk), gk + 1 and gk +3 residues from the hinge region in blue. Sticks coloured by CPK convention correspond to the residues that are affected by the G2019S and I2020T mutations segregating with PD. G2019S is part of the beginning of the activation loop. Adenosine diphosphate (ADP) (in stick representation and coloured by CPK convention) and the Mg2+ (depicted as a green sphere) ion are shown in the active site. In the right panel, the LRRK1 and LRRK2 homology models are overlayed. LRRK2 is colour coded as in the left panel and LRRK1 is depicted in black. The extra loop found in LRRK1 and not in LRRK2 is shown.

| A  HsLRRK2_KIN  HsLRRK1_KIN  HsLRRK2_KIN  HsLRRK1_KIN  HsLRRK2_KIN  HsLRRK1_KIN  HsLRRK2_KIN  HsLRRK1_KIN  HsLRRK2_KIN  HsLRRK1_KIN  HsLRRK2_KIN  HsLRRK1_KIN | 10 20 30 40 50 60  QPRLTIPISQIAPDLILADLPRNIMLN*NDELE*FEQAPEFLLGDGSFGSV-YRAAYEGEEV  HPDLPVPLQELVPELFMTDFPARL*FLENSKLE*HSEDEGSVLGQGGSGTVIYRARYQGQPV  * * +*+ ++ *+* + * * + * * +** + +** * *+* *** *+*+ *  70 80 90 100 110 120  AVKIFN-----------KHTS*LRLLR------------QELVVLCHL*HHPSLISLLAAGI  AVKRFHIKKFKNFANVPADTMLRHLRATD*AMKNFSEFRQEASMLHA*LQHPCIVALIGISI  *** *+ * ** ** ** +* * ** +++*+ *  130 140 150 160 170 180  RPRMLVMELASKGS*LDRLLQ*QDKAS-----LT*RTLQHRIALHVADGLRYL*HSAMIIYRDL  HPLCFALELAPLSS*LNTVLS*ENARDSSFIPLG*HMLTQKIAYQIASGLAYL*HKKNIIFCDL  * +*** **+ +* ++ * * +** +* ** *** **+ **  190 200 210 220 230 240  KPHNVLLFTLYPNAAIIAKIADYGIAQYCCRMGIKTSEGTPGFRA*PEVAR*GNVIYNQQAD  KSDNILVWSLDVKEHINIKLSDYGISRQSFHEGALGVEGTPGYQA*PEI-R*PRIVYDEKVD  * *+*+++* * *++****++ * *****++***+ * ++*+++ *  250 260 270 280 290 300  VY*SFGLLLYDIL*TTGGRIVEGLK*FPNEFDELEI*QGKLPDPVKEYG*CAP----WPMVEKLI*  *MFSYGMVLYELLS*-GQRPALG------HH*QLQIAKKLSKG*IRPVLGQPEE*VQFRRLQALM*  ++*+*++**++*+ * * * +*+* ** ++ * + ++ *+  310 320  *KQCL*KENPQERPT*SAQVFDILNS*AELVC  *MECWD*TKPEKRPL*ALSVVSQMKD*PTFAT  +* *++** + * + |
| --- | --- |

B


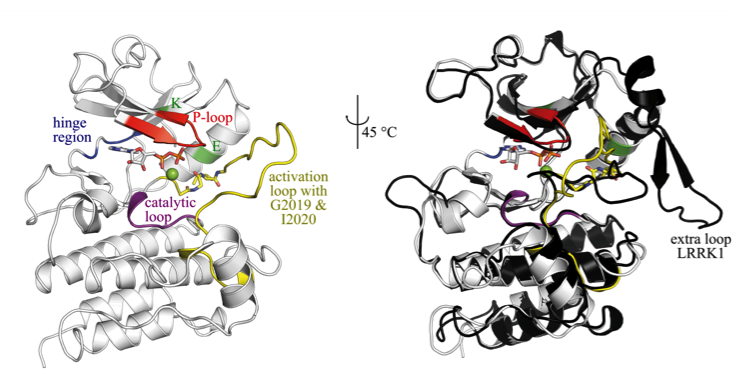

Supplement: Figure S5 — Sequence alignment and structure comparison of LRRK1 and LRRK2 KIN domains. (DOCX) [file pone.0043472.s005.docx]
