# Supplementary material for: Biochemical Characterization of Highly Purified Leucine-Rich Repeat Kinases 1 and 2 Demonstrates Formation of Homodimers
Source: PLoS One. 2012 Aug 29;7(8):e43472. doi: 10.1371/journal.pone.0043472 (PMC3430690; doi:10.1371/journal.pone.0043472)

**Figure S6.**

ATP binding of LRRK1 and LRRK2 to ATP is not disrupted in the presence of 1 mM GTP. ATP binding was tested for both LRRK1 and LRRK2 by affinity binding of the proteins to 6-AH-ATP-A or 8-AH-ATP-A-bound agarose beads.


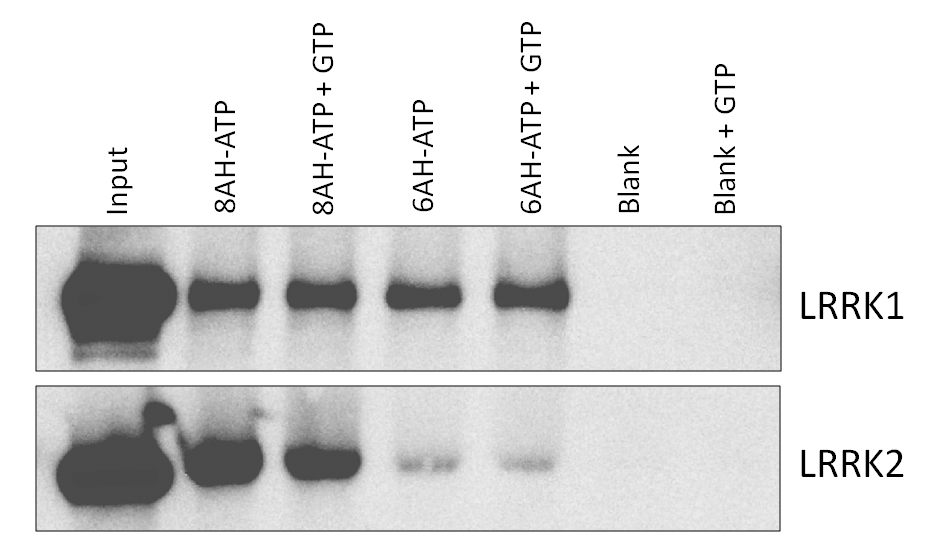

Supplement: Figure S6 — ATP binding of LRRK1 and LRRK2 to ATP is not disrupted in the presence of 1 mM GTP. (DOCX) [file pone.0043472.s006.docx]
