# Supplementary material for: Biochemical Characterization of Highly Purified Leucine-Rich Repeat Kinases 1 and 2 Demonstrates Formation of Homodimers
Source: PLoS One. 2012 Aug 29;7(8):e43472. doi: 10.1371/journal.pone.0043472 (PMC3430690; doi:10.1371/journal.pone.0043472)

**Figure S7.** Immunogold EM analysis of 3xFlagLRRK2 compared with a pre-adsorbed sample as negative control. Purified LRRK2 wild-type was labeled with (A) anti-Flag M2 monoclonal mouse primary antibodies pre-absorbed with Flag peptide followed by gold-conjugated secondary anti-mouse antibodies and (B) with anti-Flag M2 monoclonal mouse primary antibodies and gold-conjugated secondary anti-mouse antibodies. The majority of the signal is abolished when pre-absorbed antibodies are used. Image in (A) appears darker than (B) because fewer gold particles (electron dense) are present and the contrast is assigned automatically by the software. Bar is 50 nm for both images.


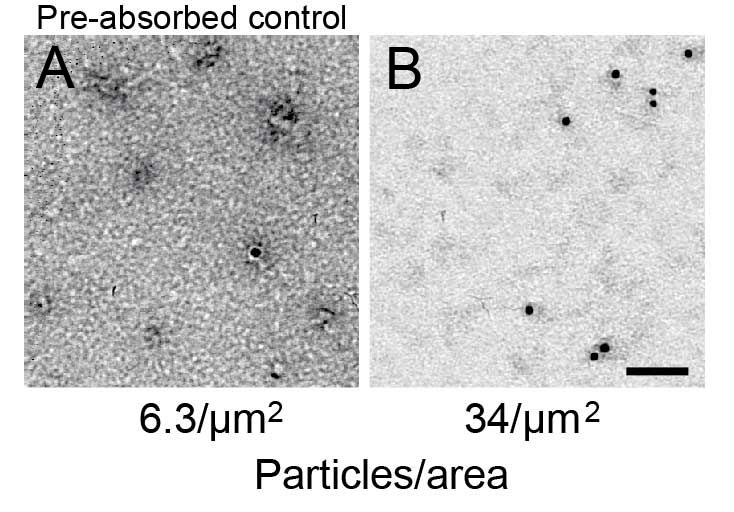

Supplement: Figure S7 — Immunogold EM analysis of 3xFlagLRRK2 compared with a pre-adsorbed sample as negative control. (DOCX) [file pone.0043472.s007.docx]
