# Supplementary material for: Biochemical Characterization of Highly Purified Leucine-Rich Repeat Kinases 1 and 2 Demonstrates Formation of Homodimers
Source: PLoS One. 2012 Aug 29;7(8):e43472. doi: 10.1371/journal.pone.0043472 (PMC3430690; doi:10.1371/journal.pone.0043472)
Supplement: Figure S8 — Analysis of frequency distribution of distances between particles in the presence or absence of 6M GdHCl for LRRK1 and LRRK2 wild-type. (DOCX) [file pone.0043472.s008.docx]

**Figure S8.** Analysis of frequency distribution of distances between particles in the presence or absence of 6M GdHCl for LRRK1 and LRRK2 wild-type. Distances between couples of particles greater than 200 nm (2000 Å) were not considered in the analysis. Distances were weighed by the area of the concentric annulus with a difference in radius that correspond to the bin size (2.5 nm). Addition of GdHCl dramatically reduces the number of dimers.

**
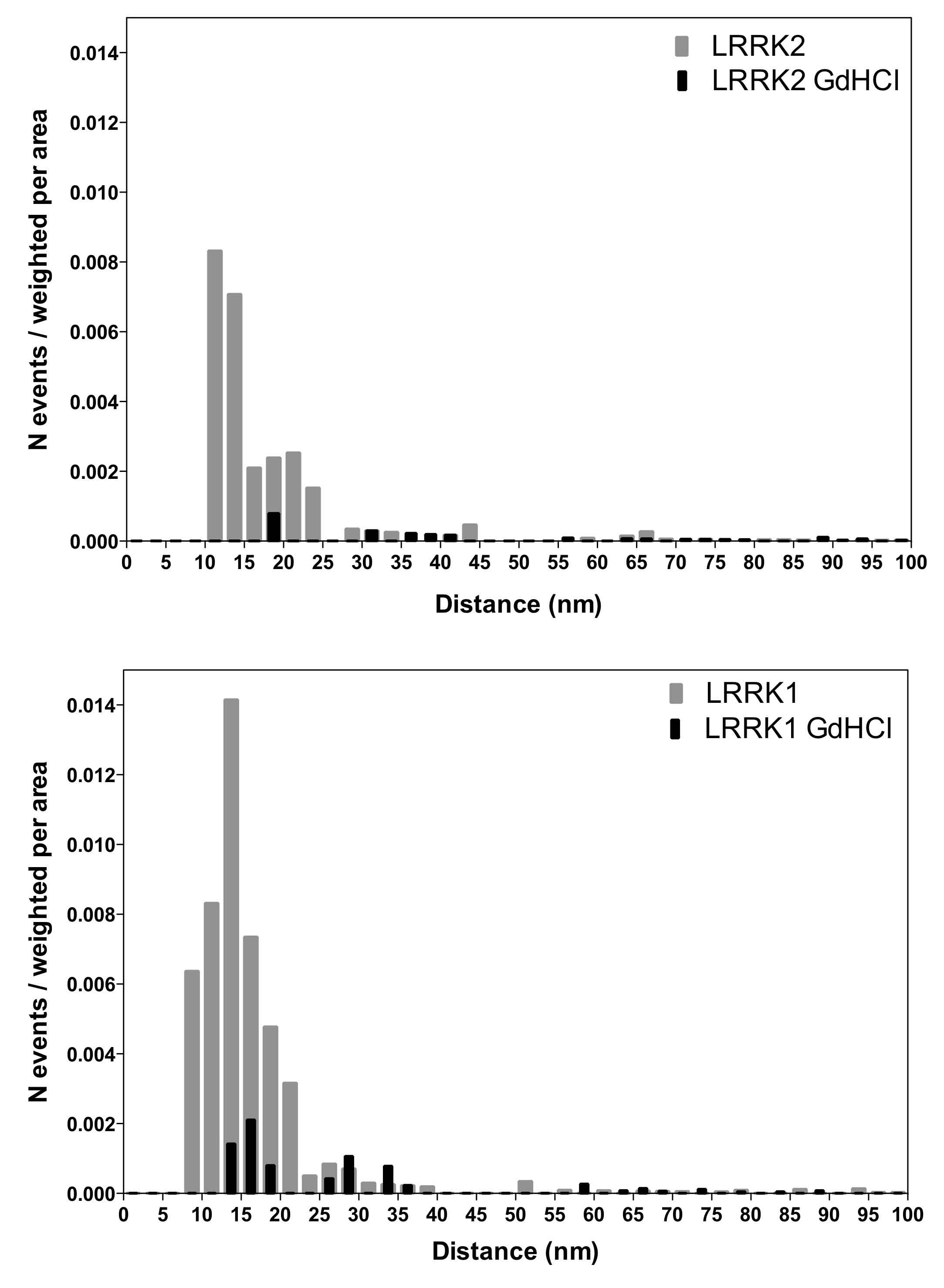
**
