# Supplementary material for: Biochemical Characterization of Highly Purified Leucine-Rich Repeat Kinases 1 and 2 Demonstrates Formation of Homodimers
Source: PLoS One. 2012 Aug 29;7(8):e43472. doi: 10.1371/journal.pone.0043472 (PMC3430690; doi:10.1371/journal.pone.0043472)

**Figure S9.** Analysis of different LRRK1 variants by immunogold EM reveals existence of dimeric proteins both in cell lysates and purified samples. Distributions of gold particle distances of gold-labeled LRRK1 wild-type (A), kinase dead K1269M (B) and GTP deficient binding mutant K650A (C). Purified proteins are shown in black-bin histograms and lysates in gray-bin histogram (inset graphs). Distances between particles were measured within 200 nm and weighed by the area of the annulus of thickness correspondent to the bin size (2.5 nm). Refer to the materials and methods section for a more detailed explanation of the analysis.


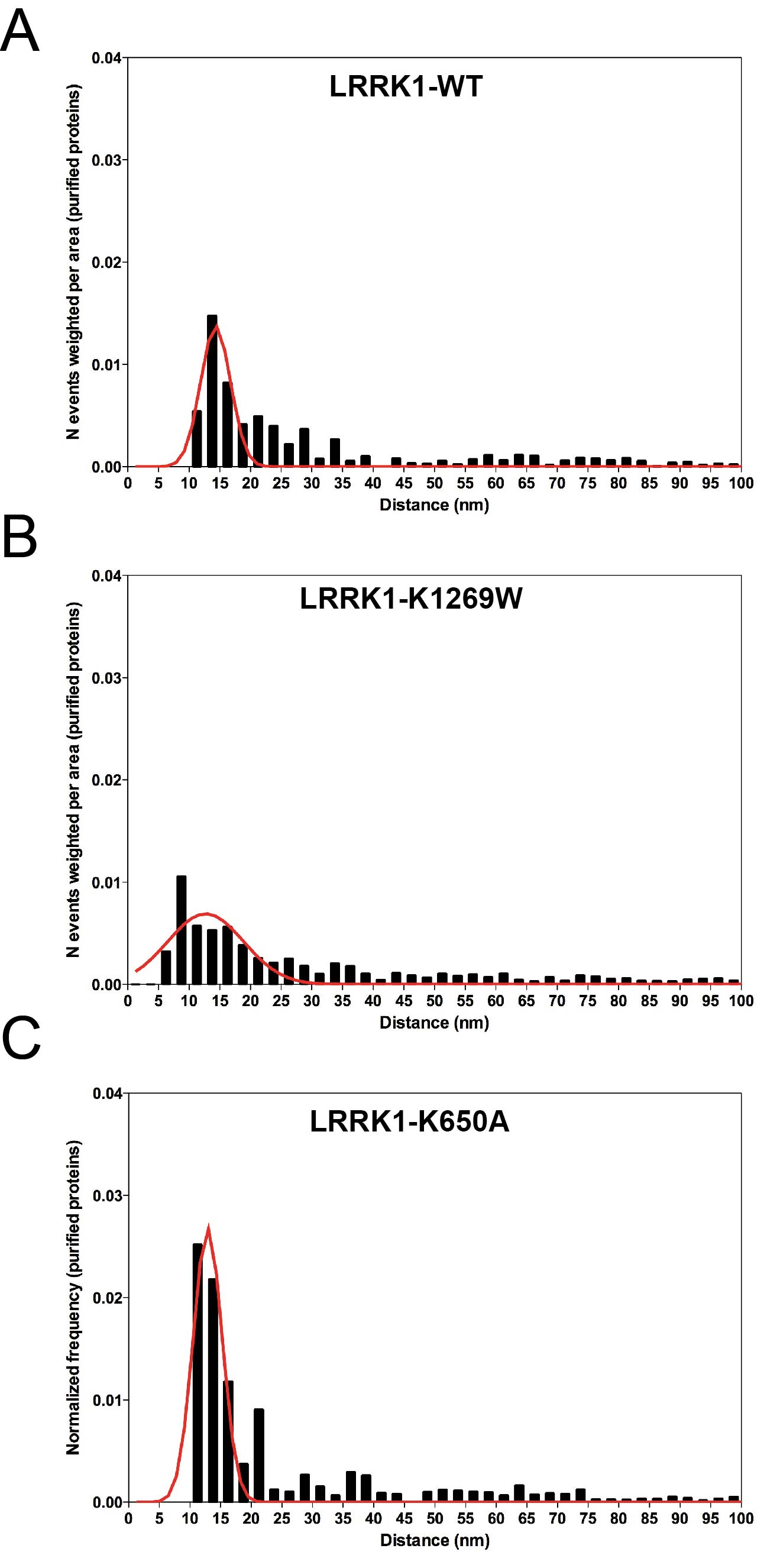

Supplement: Figure S9 — Analysis of different LRRK1 variants by immunogold EM reveals existence of dimeric proteins both in cell lysates and purified samples. (DOCX) [file pone.0043472.s009.docx]
