# Supplementary material for: Biochemical Characterization of Highly Purified Leucine-Rich Repeat Kinases 1 and 2 Demonstrates Formation of Homodimers
Source: PLoS One. 2012 Aug 29;7(8):e43472. doi: 10.1371/journal.pone.0043472 (PMC3430690; doi:10.1371/journal.pone.0043472)

**Figure S10.**

Size exclusion chromatography profiles of standard calibration molecules. The table indicates the elution volume of each standard.


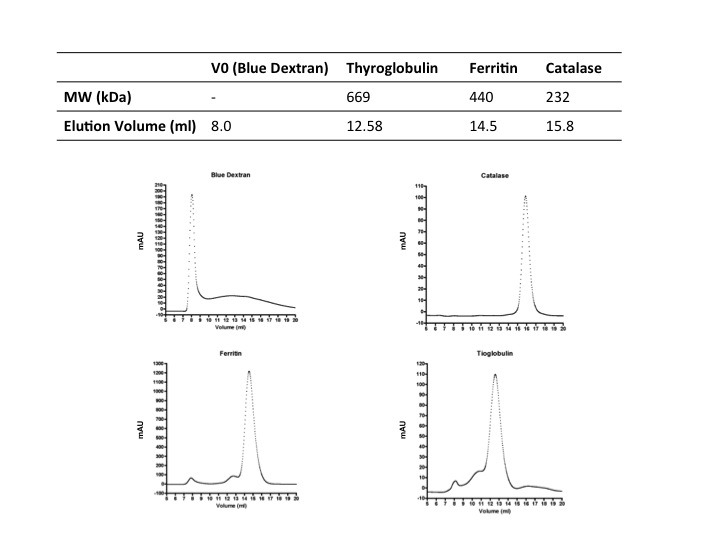

Supplement: Figure S10 — Size exclusion chromatography profiles of standard calibration molecules. The table indicates the elution volume of each standard. (DOCX) [file pone.0043472.s010.docx]
