# Supplementary material for: Biochemical Characterization of Highly Purified Leucine-Rich Repeat Kinases 1 and 2 Demonstrates Formation of Homodimers
Source: PLoS One. 2012 Aug 29;7(8):e43472. doi: 10.1371/journal.pone.0043472 (PMC3430690; doi:10.1371/journal.pone.0043472)

**Figure S11.**

Western blot of NIH3T3 total lysate and 12.5 ml chromatographic fraction against known LRRK2 interactors. The presence of endogenous beta-tubulin, Hsp90 and 14-3-3 pan could not be detected in the 12.5 ml chromatographic fraction using a 1:1000 dilution of the antibodies and 30 minutes film exposure.


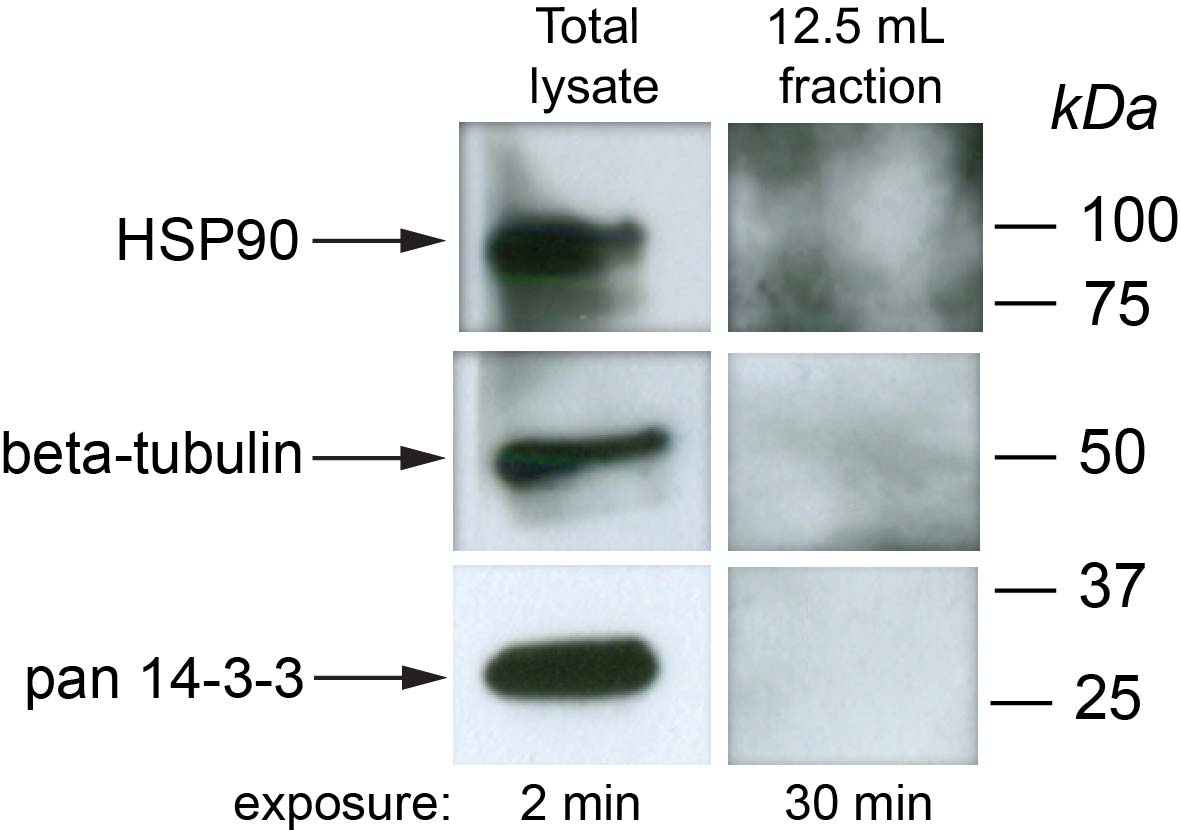

Supplement: Figure S11 — Western blot of NIH3T3 total lysate and 12.5 ml chromatographic fraction against known LRRK2 interactors. (DOCX) [file pone.0043472.s011.docx]
